# Living Wild in a Mediterranean Island: Spatial and Temporal Behaviour of Free-Roaming Cats in Cyprus

**DOI:** 10.3390/ani16071101

**Published:** 2026-04-03

**Authors:** Michalis Zacharia, Ioannis N. Vogiatzakis, Savvas Zotos

**Affiliations:** 1 Faculty of Pure and Applied Sciences, Open University of Cyprus, 89 Yiannou Kranidioti Avenue, Latsia 2231, Cyprusioannis.vogiatzakis@ouc.ac.cy (I.N.V.); 2 Department of Forests, Ministry of Agriculture, Rural Development and Environment, 26 Louki Akrita, Nicosia 1414, Cyprus; 3 Department of Soil, Plant and Food Sciences, University of Bari Aldo Moro, 70126 Bari, Italy

**Keywords:** core activity, daily activity, *Felis catus*, feral, GPS tracker, home range, locomotion, stray, urban ecology

## Abstract

Cats are among the most beloved and affectionate companion animals to humans. Historically, they have been utilised to manage pests or offer comfort and companionship, a practice that continues today. Due to human malpractice, unowned free-roaming cats (as stray pets or feral cats) are now considered amongst the 100 worst invasive species, and are responsible for the decline and even the disappearance of many wild species worldwide. Free-roaming cats maintain their hunting instincts, causing problems for native species, which is recognised as a major issue in island biodiversity. Despite their impact, limited studies have been conducted to understand the spatial activity of free-roaming cats in the Mediterranean when they are away from their caregivers (owners who feed and care for their cats while allowing unrestricted outdoor roaming). To investigate this, we used GPS tracking collars to monitor 15 free-roaming cats on the island of Cyprus, during spring–autumn 2022. The monitored cats were active in a spectrum of different habitats, from forests and farmland to shrublands and the suburbs. We monitored cats for 5.6 days, on average, to investigate their home range sizes (KDE 95%; median: males = 55,678 m^2^; females = 11,377 m^2^), daily distance travelled (median: males = 1233 m; females = 538 m), and daily/nocturnal activity, and the factors that influence these patterns. The animals’ sex, shelter availability, and the type of coverage in an area show statistically significant differences in relation to their home range, while activity peaked during the afternoon hours, a finding that is also statistically confirmed. Although the sample size of the study is relatively small, the influence of environmental and anthropogenic factors on the home range of free-roaming cats in Cyprus is revealed. These findings offer quantitative evidence and can contribute to wildlife conservation and free-roaming cat management.

## Simple Summary

Domestic cats are valued greatly as companion animals. Despite this, when allowed to roam freely outdoors, their natural hunting behaviour can significantly threaten wildlife, harming native species, particularly in island ecosystems. Despite this concern, relatively few studies have examined the spatial activity of free-roaming cats in the Mediterranean region. This study investigates the movement pattern of 15 free-roaming cats in Cyprus using GPS tracking collars to examine how different factors affect their movements. Cats were monitored for an average of 5.6 days across a variety of habitats, including forests, agricultural land, shrublands, and suburban areas. Results showed that cats travelled an average of 1.002 km per day and had measurable home ranges (KDE 95% = 36,088 m^2^). Activity levels peaked during the afternoon. Factors such as sex, shelter availability, and land cover type significantly influenced home range size and movement patterns. Although the sample size was limited, the findings provide quantitative evidence on how environmental and human-related factors shape free-roaming cat behaviour, contributing to improved wildlife conservation and cat management strategies.

## 1. Introduction

Domestic cats are among the most popular pets worldwide, and the most common pet across Europe. Their numbers have contributed to the increase in the European domestic animal population over the last decade, which now stands at 113.59 million [1]. Cats have occupied important roles in human lives, living alongside us for thousands of years [2]. Domestication began when cats approached humans to exploit their settlements for food sources, such as rats and other pests [3]. The resilience of cats in different environments, combined with their usefulness in removing harmful species, created suitable living conditions enabling them to gradually diverge from their “wild” relatives [2].

Despite their domestic nature, cats are often allowed to roam freely outside their homes. Free-roaming cat populations may include individuals described as stray or feral, although the use of these terms varies in the literature [4,5]. In this study, we use the term free-roaming cats to refer to cats that move freely outdoors, regardless of their ownership status or level of socialisation. Allowing cats free outdoor access can, in some cases, negatively affect their welfare [6] as it increases the risk of injury or illness [7], thus potentially reducing their quality of life and life expectancy [8]. Free-roaming cats can establish themselves in the wild, but their presence is highly linked with human settlements [9].

These behaviours not only affect the cats’ health but also have a significant impact on local wildlife. They live independent from prey availability [10], but still retain their hunting instincts [11] and are extremely efficient predators by nature [12]. Their presence can have a significant negative impact on wild fauna ([5,13] but see [14]).

Numerous studies have dealt with the impact of free-roaming cats on wild fauna, almost unanimously concluding that free-roaming cats are a threat to biodiversity [15,16]. In the past few years, targeted research revealed the negative impact of feral cats, causing population declines in various species [17,18,19]. In several cases, the presence of cats has been identified as the main factor for the dramatic population decline of protected species, such as *Bettongia penicillata* [20], or *Myrmecobius fasciatus* [21,22], or for the extinction of protected species such as the Desert rat-kangaroo (*Caloprymnus campestris*) [23].

Often, free-roaming cats prefer to utilise a narrow but well-defined Home Range—HR [16] (HR is the area in which an individual engages in its normal activities and that provides the necessary resources to meet its biological needs [24]. The extent of this home range plays an important role in wildlife predation [25,26] since cats, due to their opportunistic foraging behaviour, hunt and consume what is available within this area [15]. The extent of the home ranges varies depending on environmental factors (e.g., habitat type, vegetation) and cats’ attributes (e.g., age and sex) [27]. In comparison with areas within the urban fabric, the home ranges of free-roaming cats seem to be wider in rural areas [28] or forested areas [16,29]. In areas with low housing densities, cats need to travel greater distances in search of food or prey [30]. The continuous supply of food to free-roaming cats expands the problem by giving them additional resources, resulting in increased density [31], which in turn affects the local fauna [32,33], even within inhabited areas [34].

In Cyprus, the relationship between cats and humans dates back to ancient times. Cyprus is home to the oldest discovery of a buried cat alongside its caretaker, dating to around 9500 years ago [35,36]. Since Cyprus has never been connected to another continent, it appears that the species arrived on the island through human intervention [37]. It is suggested that cats were introduced to the island from Asia between 11,000 and 9000 BCE, in order to combat rodents that were causing problems for crops [38]. Regardless of this historic relationship, the ecological impact of free-roaming domestic and feral cats on wildlife remains understudied in some regions, and Cyprus is no exception.

Free-roaming cats in Cyprus have never been censused, and therefore their overall population size and distribution are unknown. The majority of the environmental and animal welfare organisations in Cyprus recognise an informal approximation of around a million (Cyprus Veterinary Services, personal communication), stating that the species can be found all over the island in a stray/feral state. If the above estimate is correct, then the species population density is approximately 108 per km^2^, which is comparatively higher than the density of cats in the districts of New York [39] or Texas [40], where densities of 35.9 and 32 cats per km^2^, respectively, have been recorded, or Windsor, Ontario, with a similar size to Nicosia (13.3 cats per km^2^) [41]. Currently, animal welfare organisations in Cyprus are trying to provide, when/where possible, food and shelter in city parks or to collaborate with citizens who maintain small feeding stations near their properties.

A large portion of free-roaming cats seems to form colonies, finding shelter in abandoned houses and feeding themselves with food remains in public trash bins. This creates problems not only for the animal’s welfare and the local fauna, but also for human health and safety [42,43]. More than 1000 free-roaming cats are reported as roadkill each year [44], while recently, a Feline Infectious Peritonitis (FIP) (a severe and invariably fatal disease affecting both domestic and wild felines with limited effective therapeutic options available) outbreak occurred in Cyprus. More than 10,000 cases were presented in the veterinary clinics from January 2023 until July 2023 due to clinical signs of FIP. The high number of free-roaming cats in Cyprus is something that potentially played a key role in this outbreak [45]. Therefore, high density combined with mobility increases health and ecological risk.

Monitoring the movement and spatial ecology of free-roaming cats is essential for understanding how their population interacts with urban environments and wildlife. In recent years, advances in GPS telemetry have enabled researchers to track individual animals with high spatial and temporal accuracy, providing detailed information on movement behaviour and activity patterns, thus contributing to the understanding of species ecology and conservation [46,47]. However, location fixes may contain a certain degree of error related to device reliability, terrain morphology, and the habitat in which the animal moves.

An additional limitation concerns the fitting of the collar and the weight of the device, as these may influence the natural behaviour of the animal [48]. The limitation on device weight often results in shorter battery life, and consequently shorter monitoring duration and fewer data points per animal [49], which may limit the quantity and resolution of the available data [26]. High cost, potential technical failures, the difficulty of identifying suitable individuals for monitoring, as well as the difficulty of recapturing animals for device retrieval or redeployment, are factors that often lead to smaller sample sizes and limited datasets in telemetry studies [46].

To reduce these disadvantages, various methodological measures are taken, such as fully charging the devices prior to deployment, as well as appropriately adjusting recording parameters in order to achieve energy efficiency without compromising data quality, for example through the use of energy-saving functions (sleep mode) or by adjusting the location fix frequency. Additionally, tests are conducted to assess the accuracy of location fixes within the study area [48], while the total weight of the equipment (including GPS and collar), when monitoring small mammals such as cats, should not exceed the acceptable limit, which is <5% of the animal’s body weight [50].

Despite the increasing use of GPS telemetry in studies of free-roaming cats in other regions, similar research has not been conducted in Cyprus. Consequently, there is very limited information on the movement ecology, spatial behaviour, and habitat use of the island’s large feral cat population. This study aims to address this gap by deploying GPS collars on free-roaming cats to:

(a) estimate the extent of free-roaming cats’ home range on the island;
(b) identify the parameters that may influence the species’ spatial ecology (e.g., sex, shelter/feeding availability, land-cover composition);
(c) analyse free-roaming cats’ activity, including travel distance (i.e., morning–daytime–evening).

## 2. Methods

### 2.1. Study Area

The current study was conducted in Cyprus, the third largest island (9251 km^2^) of the Mediterranean Sea. Cyprus has a human population of nearly a million and is situated in the south-eastern part of the Mediterranean Sea [51]. Cyprus is dominated by two main mountain ranges, Troodos (max. alt. 1952 m) and Pentadaktylos (max. alt. 1024 m) separated by the flat central plain of Mesaoria in between [52]. The island has a Mediterranean climate with mild rainy winters and hot, dry summers. The annual precipitation ranges from 300 mm at the central plain to 1100 mm at the summit of Troodos Mountain range [53].

Cyprus is an island characterised by high levels of endemism [54], within the Mediterranean global biodiversity hotspots [55,56]. This is induced by its long isolation, its diversity of landscapes and geological formations [55], as well as the interaction between natural and human-induced processes such as cultivation and grazing [57]. There are 22 different species of reptiles and three amphibians in Cyprus. Amongst them are three endemic lizards, one endemic snake, and two endemic frogs [58]. Cyprus also hosts 30 species of terrestrial mammals, including two endemic rodents (*Mus cypriacus*, *Acomys nesiotes*) [59]. More than 400 species of birds have been recorded in Cyprus [60], including three endemic species and three endemic subspecies [61]. Cyprus is also an important migratory route for birds with more than 200 species considered common migrants [62]. It is estimated that 100 million birds pass through Cyprus during spring migration and 150 million birds during autumn migration [60].

**Figure 1:**
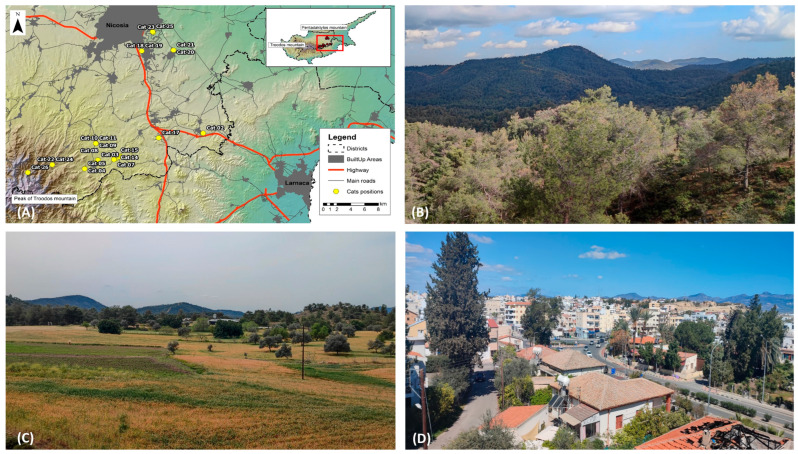
Map of Cyprus with indication of the sites where free-roaming cats were captured and monitored (**A**). Representative pictures from different land cover types where free-roaming cats were monitored. (**B**) Coniferous forest, (**C**) Agricultural area and (**D**) Urban fabric.

The present study was carried out in the Nicosia district. We tried to evenly collect and monitor free-roaming cats from different land cover types, namely forests (four cats), agricultural areas (five cats), and urban fabric (6 cats) (Figure 1 and Supplementary File S1). Forests in the study area are dominated by pines (*Pinus brutia*), accompanied by a mixture of evergreen-sclerophyllous shrubs and maquis vegetation. The agricultural area of the study is characterised by small to medium-sized parcels of arable fields (mainly cereals) with the presence of hedgerows of shrubs and small trees. The urban fabric consisted of residential areas near the city (suburbs) with mainly detached houses. City parks and green spaces are abundant but not well managed, while streets have relatively low traffic volume in relation to the centre of the city.

### 2.2. Capture and Monitoring Procedures

A total of 15 free-roaming cats were successfully captured and monitored between March and October 2022. Each cat was captured in a different area (see Figure 1 and Supplementary File S2) to avoid possible overlap. Of the total cats monitored, six were females (40%) (one neutered), and nine were males (60%) (all unneutered). Only nine cats were using a regularly maintained feeding station (shelter). The cats were monitored using “A21P GPS tracker” (Manufacturer: Shenzhen I365-Tech Co., Ltd; City: Shenzhen, China; Sourced: https://www.a-store.gr, Karditsa, Greece), a lightweight (40 g; 57 g with collar and protective casing), small-sized (5.0 cm × 2.7 cm × 1.7 cm), low-cost (c. 80–100 euros per unit) GPS unit, usually used by owners of domestic cats. The battery of the devices (1000 mAh), when enabling the sleep mode features (i.e., saving energy when the animal is resting) to extend the battery life, allowed us to collect fixed locations every 10 min. This interval period is similar to other studies: every 5 min [63] and every 15 min [64,65]. The mean tracking period was 5.6 days on average (in hours: average = 136, min–max; 63–218), following studies with a similar approach [66,67,68], while the mean number of GPS fixes (the single location recorded by the GPS device at a given time) was 208 (min–max: 95–386) (see Supplementary File S1). Despite the limited monitoring duration, graphs showing the cumulative size of the HR area against the number of GPS fixes (Supplementary File S2) suggest that sampling effort is sufficient for reliable HR estimation. We acknowledge that the relatively short tracking period represents an important limitation of this study. This is an inherent challenge when monitoring small- to medium-sized animals with GPS collars recording frequent location fixes. While the data may reflect short-term activity patterns rather than fully stable long-term home ranges, they provide valuable ecological insights into the spatial behaviour of free-roaming cats and are able to guide future research and management strategies [see 26, 63, 66, 67].

**Figure 2:**
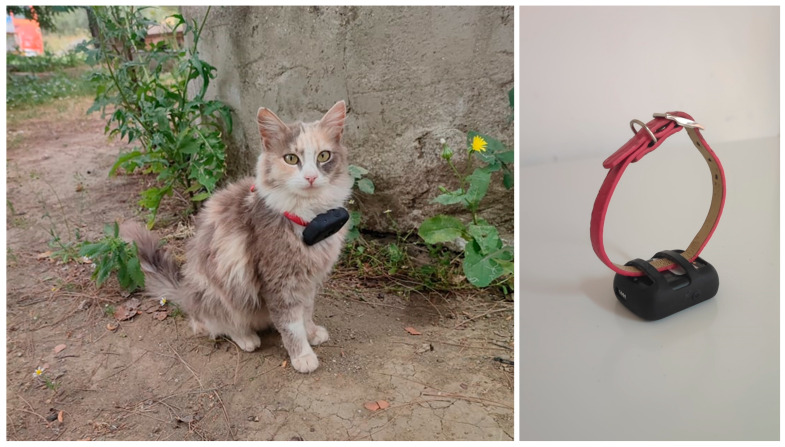
(**Left**): picture of a free-roaming cat (code 11), bearing an A21P GPS tracker. (**Right**): close-up of the A21P GPS tracker with the hard plastic collar used to firmly attach the GPS casing to the cats, avoiding removal.

A hard plastic collar acquired from a pet shop was used, instead of the soft fabric one, to firmly attach the GPS casing to the cats and avoid removal (Figure 2). To minimise the potential risks to the animals (e.g., snagging, entanglement, strangulation), all cats were recaptured at the end of the monitoring period, and the GPS collars were removed. In addition, the animals’ locations were continuously monitored throughout the study to allow prompt intervention in the event of abnormal movement patterns or potential welfare concerns. The 5% rule for animal-borne devices [50,69], was strictly followed. The rule states that for animal welfare purposes, no monitoring devices attached to the animal should weigh more than 5% of the animal’s weight. In the case of our study, the lighter cat was approximately 2 kg, and the GPS collars should respect the maximum weight threshold of 100 g (2.000 g × 5%).

For the majority of our monitored animals (*n* = 12), capturing and placement of the GPS collar was conducted with the help of the person feeding the cats. Less manageable feral cats (*n* = 3) were captured with Tomahawk traps [70], and were transferred to a veterinary clinic, where the attachment and removal of the GPS collars were performed by a veterinarian following proper sedation and health assessment protocols. Approximately half of the animals showed brief discomfort behaviour after the placement of the collar, which, however, diminished and even disappeared after a few minutes. The absence of an acclimatisation period, due to the limited battery life of the GPS, is an important limitation of the study that might have influenced the behaviour of the animals. All procedures were conducted under consideration of animal welfare and ethical aspects and with the approval of the Cyprus Veterinary Services (26 May 2022) (AF: 02.05.001.003.037/15) and the Cyprus bioethics committee (18 April 2022).

After capturing the animals, the following characteristics were recorded per individual: weight (to the nearest g), sex (male/female), age class (young/mature—based on age stages proposed by Quimby et al., (2021) [71]), and presence of shelter in the form of a wooden structure where the animal could find shelter and food (yes/no). Shelters were either managed by animal welfare organisations or by area residents. Individuals were sampled based on capture feasibility during fieldwork.

### 2.3. Spatial Analysis

**Figure 3:**
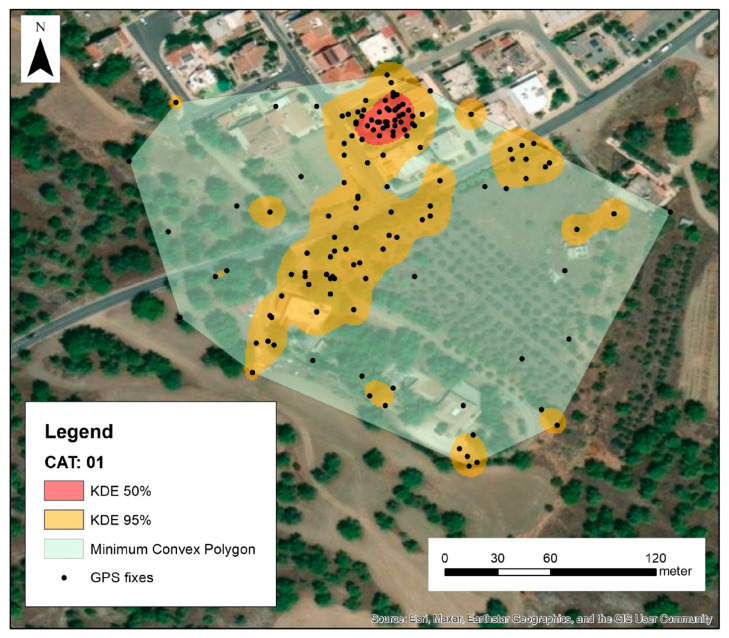
Example of home range estimation for cat #01. The core area of the home range (KDE 50%) is close to the house that provided shelter and food to the cat. The activity of the cat expands to nearby houses, while the Minimum Convex Polygons include a large percentage of agricultural land and wild vegetation.

The GPS fixes for each animal were plotted in ArcMap 10.7 [72], and false indications, common in the case of a low satellite signal, were removed. We used Minimum Convex Polygon (MCP) to produce animals’ home ranges (100%) and Kernel Density Estimation [73,74,75] to produce home (95%) and core (50%) ranges for each cat based on their recorded GPS fixes (Figure 3).

Using the “Adehabitat” package [76] in R v4.3.1 [77], the trajectories for each cat were calculated. The animals’ trajectories were used to estimate the cumulative size of the Home Range area (MCP100%) against the number of GPS fixes, as well as the meters travelled in an hour (a proxy of the animals’ activity) during different periods of the day. In addition, trajectories were utilised to study the movements of the animal within its home range by identifying:

(i) maximum linear distance travelled from the feeding station,
(ii) daily distance travelled (24 h; midnight to midnight),
(iii) distance travelled during morning hours (first activity of the day until dawn),
(iv) distance travelled during daytime (dawn to dusk),
(v) distance travelled during evening hours (dusk until the last activity of the day)

In each case, the average, minimum, maximum, and standard deviation of the distances initially travelled by each animal were calculated.

To assess the possible impact of the surrounding environment on the activity of the free-roaming cats, we calculated the percentage (%) of land cover for the main categories (forest, agricultural, and urban fabric) within the home range (MCP 100%) of each individual. Identification was based on satellite images of the World Imagery basemap [72], coupled with field observations to increase accuracy.

### 2.4. Statistical Analysis

The Shapiro–Wilk normality test was used to identify the distribution stage of our data and determine the type of subsequent statistical analyses [78]. Our data did not follow a normal distribution, and so the statistical differences between the values of two groups (e.g., sex) were assessed using the non-parametric Mann–Whitney U test [79]. In the case of more than two groups (e.g., activity per period of the day), the Kruskal–Wallis H-test was used with post-hoc analysis from Dunn’s z-test. To further examine relations between variables, linear regression analysis was applied. All statistical analyses were conducted in R Studio [80] using R [77].

## 3. Results

**Figure 4:**
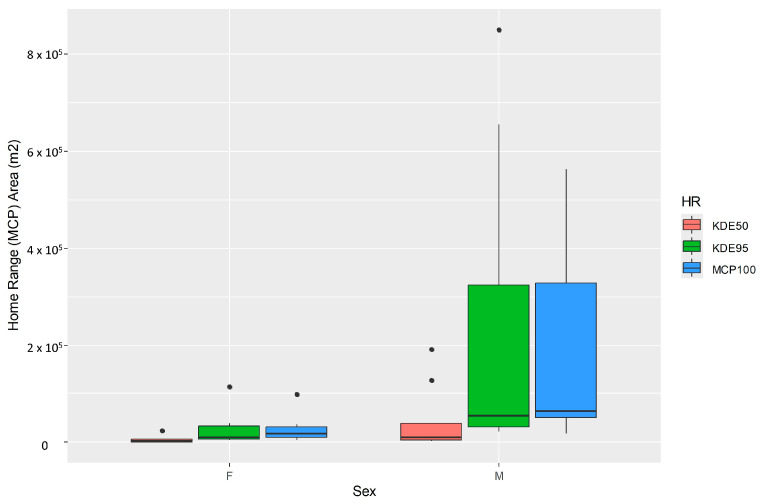
Area of home range (MCP) of cats in relation to their gender (sex).

**Figure 5:**
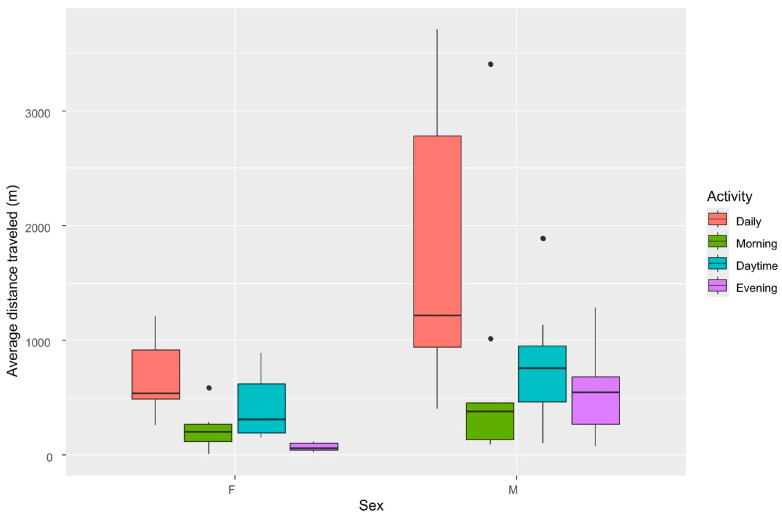
Average distance travelled as a proxy for cats’ activity, in relation to gender (sex). Average distance is presented for three main periods of the day (morning: first activity of the day until dawn; daytime: dawn to dusk; evening: dusk until the last activity of the day) as well as daily (24 h starting midnight).

**Figure 6:**
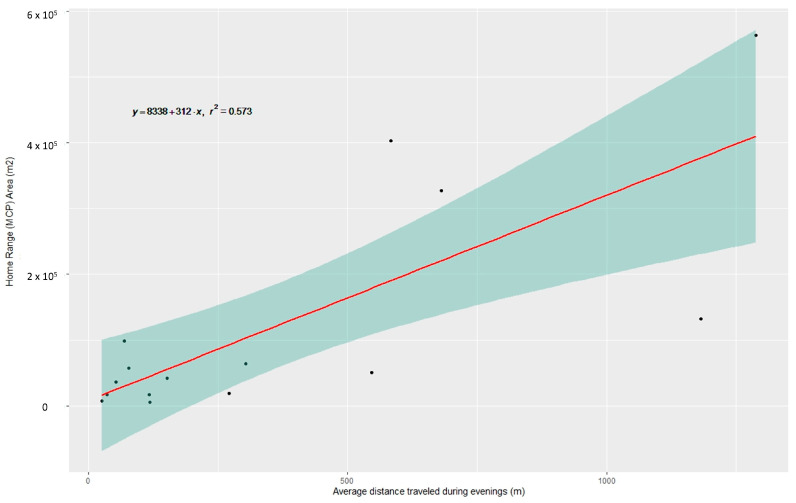
Linear relation and confidence interval between the area of home range, as estimated through Minimum Convex Polygon, and the average distance travelled during evenings (m).

Sex was a significant factor affecting HR, with male cats having significantly larger home ranges (Figure 4) (Mann–Whitney U test: *p* < 0.05) and being more active than females (Mann–Whitney U test: *p* < 0.05), covering larger distances per day (Table 1). Male cats cover on average 163.87 m/hour, in contrast to females, which cover 69.67 m/hour. A male cat having a home range (HR) around 0.05 km^2^ (max ~1 km^2^) can cover daily distances of nearly 1.5 km (max ~4 km), while females have significantly smaller HR around 0.01 km^2^ and cover shorter distances within the day (average ~500 m, max ~1.2 km; see Table 1). Although the difference between the daily distance covered by the two sexes is observed regardless of the period of the day (Figure 5), it is statistically confirmed during the evening hours (Mann–Whitney U test: *p* < 0.01). Our evidence shows that cats with larger HRs cover larger distances during the evening hours (Figure 6) (F_(1, 13)_ = 17.47, *p* < 0.01, R^2^ = 0.573). When examining the activity of the cats during the different periods of the day (i.e., morning, daytime, and afternoon), no statistically significant differences were detected in the activity of female cats. Males’ activity is increased during morning and afternoon hours (*H*(2) = 7.19, *p* = 0.027), with the afternoon activity having a significantly higher score than daytime activity (Dunn’s test: *p* < 0.002).

**Figure 7:**
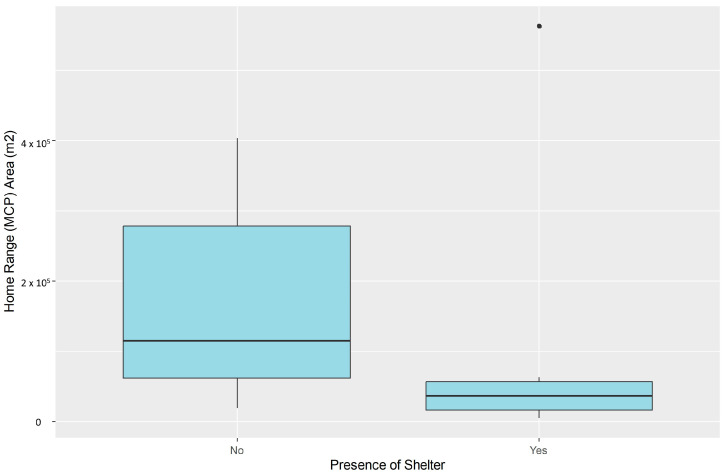
Difference in the extent of free-roaming cats’ home range based on the presence of a shelter (feeding station) within the home range.

A larger core HR area (KDE50%) also appears in cats without a shelter or feeding station (resources) regularly maintained within their home range (Figure 7). This relationship is statistically significant (Mann–Whitney U test: *p* < 0.05). The age class and the weight of the animals do not seem to have any influence on the HR of the animal.

**Figure 8:**
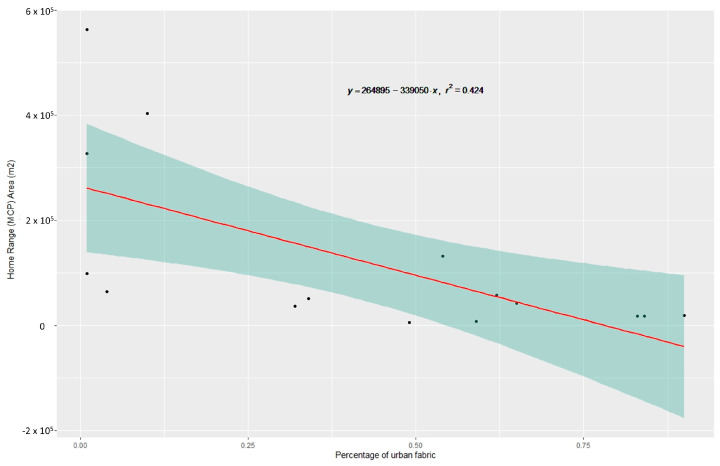
Linear relation and confidence interval between the area of home range, as estimated through Minimum Convex Polygon, and the percentage of urban fabric within that area.

Using linear regression to examine the area of the HR in relation to land cover type in that area (i.e., forest, agricultural, urban fabric), we identified significant relations only between the HR and the percentage of urban fabric. The higher the percentage of a cat’s HR covered by urban fabric, the smaller the HR of that individual (Figure 8) (MCP100: F_(1, 13)_ = 9.58, *p* < 0.01, R^2^ = 0.427; KDE95: F_(1, 13)_ = 6.63, *p* < 0.05, R^2^ = 0.338; KDE50: F_(1, 13)_ = 4.89, *p* < 0.05, R^2^ = 0.273). This indicates that free-roaming cats in urban areas tend to have smaller HR than those living in agricultural or forested areas.

## 4. Discussion

Our results demonstrate that free-roaming cats in Cyprus exhibit larger home ranges and high mobility compared to similar short-term GPS studies, highlighting the role of insular contexts and human-mediated resources in shaping spatial ecology. With an average daily distance travelled of 1000 m and an average home range (MCP 100%) of 50,440 m^2^ across all samples, the results of our study fall on the higher end of the free-roaming cats’ activity spectrum. Similar studies [26,81,82] on the home ranges of free-roaming domestic or feral cats, conducted using GPS collars over a short monitoring period of time (less than a week—Table 2), resulted in home ranges less than half of the ones observed in our study.

The large HR detected in our study might be related to the absence of competitors or predators in Cyprus, such as large carnivores, which can suppress free-roaming cats’ activity [27,83]. Although some animals in Cyprus may occasionally prey on cats, such as the red fox [84]. The absence of other large carnivores on the island allows cats to be classified as an apex predator. As a result of their ecological position, free-roaming cats can often exhibit greater spatial freedom in their movements [85,86] and expand their home ranges, unrestrained by the spatial limitations typically imposed by the presence of natural predators. Larger HRs can also be related to the favourable status that cats receive in Cyprus. Taking care of stray cats is extremely common in the island and is firmly established in local traditions and religious beliefs [87,88]. Most of the free-roaming cats in Cyprus are not pet cats, but stray cats. In contrast to the domestic cats, they are not sterile (despite efforts from the government and NGOs), and they reproduce freely. In addition to food resources obtained via hunting, free-roaming cats gain additional resources in the form of shelter, feeding stations, or availability of food waste (via trash cans) that can shape their HR patterns [89,90] and influence the HR’s size and structure [82].

The impact of those additional resources is also visible in our results, with cats enjoying feeding stations or shelter availability exhibiting smaller HR and shorter distance travelled than the cats without those resources. This trait can be linked with the Central Place Foraging (CPF) theory, where a forager takes optimal foraging decisions to maximise the energy gain per unit of time. Although our data do not directly test management interventions, additional research is needed with a larger sample size to explain what this means for both wildlife and free-roaming cats’ welfare, and if the provision of shelters could be an effective management measure for free-roaming cats. Currently, though, the presence of shelter and/or frequent feeding to free-roaming cats tends to lead to increased population densities [91], which would not contribute to resolving the broader management problem.

The results of the present study indicate a difference in home range and distance travelled by cats between sexes, with males exhibiting a larger home range and moving greater distances than females. This is a common behavioural trait [66,86,90] that has been associated partly with reproduction activity and ecological factors. Male cats tend to occupy larger home ranges during breeding season [66], to increase their breeding chances in cats’ polygamous reproductive system [89,90]. In contrast, females are gathered mainly around food resources to ensure successful gestation [30]. Considering all of the above, the factors that influence the home range of cats vary across studies, and it is difficult to determine precisely what affects the size of their home range. According to Garvey et al. (2020) [92], among other factors, the personality of the animals may also influence their overall activity, something that is important to consider in future management actions.

The monitored cats exhibited increased movement early in the morning and late in the afternoon, following patterns similar to those of other free-roaming cats elsewhere [66,93,94]. This behaviour is generally reported by free-roaming cats [95,96] and has been linked to both the reduced human disturbance during those hours [97,98] as well as to increased prey availability. In the absence of direct predation data in our study, we can only infer a similar impact. During morning and late periods, bird activity tends to be higher compared to the rest of the day [99], due to the roosting behaviour of diurnal species and the activation of nocturnal ones [100]. Evidence from studies on the predation of small mammals further supports this, revealing that small mammals experience higher rates of predation from cats during the night than during the day [101,102]. These findings highlight the ecological impact of free-roaming cats during twilight periods (dawn and dusk) when there is a greater likelihood of interactions with a wider range of wildlife species [5].

One of the key findings of this study is that free-roaming cats occupy larger home ranges in rural and forested areas than within the urban fabric. Our results are in line with the general pattern observed in cats located in forested areas [16,27,103,104], as well as cats in rural versus urban settings [28]. Smaller home ranges of cats roaming in the urban setting may be attributed to the impenetrable obstacles (e.g., house fences) often encountered in urban environments, which restrict and/or reduce cats’ movement [105]. Smaller HR in urban areas can also be related to the increased likelihood of interaction with stray or roaming dogs, common in dense housing areas, which makes cats more cautious and thus less active [30].

This can also be linked to food availability, since free-roaming cats living in forested or agricultural areas do not receive supplemental feeding by humans, or receive less than those in the city do, and can exhibit larger home ranges in search of food or prey [98].

The animals’ movement pattern on the map (Supplementary File S2) suggests that cats prefer to use natural features of vegetation and the surrounding environment to move from one location to another, such as vegetation, paths, or tree lines, while avoiding movement through open and uncovered areas, to define the boundaries of the areas they inhabit. These structures appear to allow for free and easy movement in dense vegetation, e.g., following the paths of prey in wooded areas [43], while at the same time providing cover through adjacent shrubs or tree lines, helping them to remain undetected by other predators [26]. This behaviour also has practical implications for population monitoring, as cats’ tendency to avoid open or human-made paths may make them harder to detect using camera traps.

Although, as indicated by the animals’ trajectory, the activity of most cats is concentrated around the periphery of their care and feeding areas, cats also appear to spend time in other locations, likely foraging for food [26]. Our results indicate that the majority of the cats monitored, in addition to their primary site, frequently visit at least two other areas where they are likely to be fed. Some cats may receive care from more than one caretaker. Knowing that cat population density is positively associated with human population and urban expansion [96], as well as that additional anthropogenic resources increase the HR of free-roaming cats, this opportunistic behaviour raises concerns regarding the possible increase in the impact of cats in inhabited areas and detached houses near forests, natural, or protected sites.

Understanding the impact of free-roaming cats is of particular importance in Cyprus due to its rich biodiversity [56] and its high levels of endemism [54]. Cats on the island of Cyprus are expected to face greater intraspecific and interspecific competition for resources [106], increasing their impact on wildlife. Compared to continental areas, on islands, cats consume three times more IUCN threatened and conservation concern species, with approximately 90% being birds, mammals, and reptiles [17,107]. Research conducted on islands showed that free-roaming domestic cats are the main reason for the extinction of 33 endemic vertebrates, and contributed to the decline of an additional 38 species that are now at risk of extinction [108]. By combining high-resolution GPS tracking with land-cover analyses, our study adds empirical evidence from a Mediterranean island context.

The home range area and activity patterns of the cats reveal that they move way beyond their feeding and resting sites (core HR areas). They remain for extended periods in neighbouring regions, primarily consisting of areas close to human habitation, searching most likely for food or prey. The variety of food options available to them (e.g., trash bins) likely increases their activity and generally influences their movement patterns. These patterns highlight the influence of human-provided resources on the spatial behaviour of free-roaming cats and provide a foundation for future studies on their ecology.

## 5. Conclusions

Despite the relatively small sample size of this study (15 individuals) and the limitations of the short tracking duration, this study provides new insights into the spatial ecology of free-roaming cats in Cyprus. The results show that cats on the island exhibit relatively large home ranges and high mobility compared to similar studies. These patterns appear to be influenced by the island context, including the absence of large predators, the ecological role of cats as top predators, and the widespread availability of human-provided resources such as feeding stations, shelters, and food waste. Sex, habitat type, and resource availability were all significant factors shaping movement behaviour and home range size.

The findings also highlight clear spatial and temporal activity patterns. Male cats travelled farther and occupied larger home ranges than females, likely due to reproductive strategies. Cats were most active during early morning and late afternoon periods, which coincide with reduced human disturbance and increased prey activity. Additionally, cats in rural and forested environments used larger areas than those in urban settings, while natural landscape features such as vegetation corridors and paths appeared to facilitate movement.

Overall, this study contributes empirical evidence on the behaviour of free-roaming cats in a Mediterranean island ecosystem. Understanding how environmental and human-related factors influence cat movement is important for improving monitoring strategies and informing future wildlife conservation and free-roaming cat management efforts in Cyprus and similar island systems.

## Figures and Tables

**Table 1:** Summary statistics for the 15 free-roaming cats monitored. The percentage of Land Cover Type (i.e., forest, agricultural, urban) within the HR was estimated based on MCP 100%.

	Males (*n* = 9)		Females (*n* = 6)		Total (*n* = 15)	
	Median	(Min–Max)	Median	(Min–Max)	Median	(Min–Max)
Core home range—KDE 50% (m^2^)	10,888	2059–191,391	2204	1050–23,406	6826	1050–191,391
Home range—KDE 95% (m^2^)	55,678	21,089–849,732	11,377	5021–114,033	36,088	5021–849,732
Home range—MCP 100% (m^2^)	63,950	18,602–563,140	17,417	5395–98,364	50,440	5395–563,140
Forest areas within HR (%)	20	0–72	18	0–99	19	0–99
Agricultural areas within HR (%)	33	7–85	19	0–42	26	0–85
Urban fabric within HR (%)	34	1–90	54	1–84	49	1–90
Maximum linear distance from home (m)	254	104–800	143	84–290	202	84–800
Daily distance travelled (m)	1233	404–3714	538	263–1214	1002	263–3714
Morning distance travelled (m)	377	94–3411	205	11–590	272	11–3411
Daytime distance travelled (m)	760	104–1892	311	157–895	662	104–1892
Evening distance travelled (m)	546	78–1288	61	26–118	152	26–1288

**Table 2:** Comparative home range estimates across different studies that monitored the spatial activity of free-roaming cats for a short period of time (less than a week). Home range was calculated using Kernel Density Estimation (KDE 95%, 50%) and Minimum Convex Polygon (MCP 100%, 95%).

		Average Home Range (m^2^)					
Country	Study	KDE 95%	KDE 50%	MCP 100%	MCP 95%	No. of Cats	Tracking Period
Australia	Meek 2003 [81]	-	-	22,900	-	15	7 days
China	Zhang et al. 2022 [66]	76,600	15,000	68,000	-	29	5 days
**Cyprus**	**This study**	**36,088**	**6826**	**50,440**	**-**	**15**	**6 days**
France	Philippe-Lesaffre et al. 2024 [67]	41,000	5100	-	-	55	>3 days
Norway	Bachmann 2020 [28]	47,000	6500	-	28,900	104	7 days
Ontario	Pyott et al. 2024 [26]	-	-	8000	-	42	6 days
Tabarca Island	Molina-Bernabéu and López-Iborra 2024 [82]	12,500	2400	-	-	3	7 days
UK	Hanmer et al. 2017 [68]	16,600	2300	-	11,800	38	7 days
UK	Dunford et al. 2024 [63]	-	-	86,300	-	56	4 days

## Data Availability

The original contributions presented in this study are included in the article/Supplementary Materials. Further inquiries can be directed to the corresponding author.

## Supplementary Materials

The following supporting information can be downloaded at: https://www.mdpi.com/article/10.3390/ani16071101/s1, File S1: Individual data from the 15 monitoring cats, accompanied by data on monitoring start date/time, monitoring duration, Home Range, percentage of overlapping area and distance travelled in meters. File S2: Spatial and Temporal data of the 15 monitoring cats. Data includes Trajectory maps, Time lag charts between GPS fixes, cumulative home range areas (Minimum Convex Polygon 100%) by the number of GPS fixes and Home Range maps in the form of 100% Minimum Convex Polygon as well as Kernel Density Estimation (50% for the core area and 95%).
