# Supplementary material for: Living Wild in a Mediterranean Island: Spatial and Temporal Behaviour of Free-Roaming Cats in Cyprus
Source: Animals (Basel). 2026 Apr 3;16(7):1101. doi: 10.3390/ani16071101 (PMC13072416; doi:10.3390/ani16071101)
Supplement: Supplementary file 1 [file animals-16-01101-s001.zip › Suppementary File S2.pdf]

Living Wild in a Mediterranean Island: Spatial and Temporal Behaviour of Free-Roaming Cats in Cyprus

Michalis Zacharia, Ioannis N. Vogiatzakis and Savvas Zotos

Supplementary file S2

Spatial and Temporal data of the 15 monitoring cats. Data includes Trajectory maps, Time lag charts between GPS fixes, cumulative home range areas (Minimum Convex Polygon 100%) by the number of GPS fixes and Home Range maps in the form of 100% Minimum Convex Polygon as well as Kernel Density Estimation (50% for the core area and 95%).

CAT
ID 01

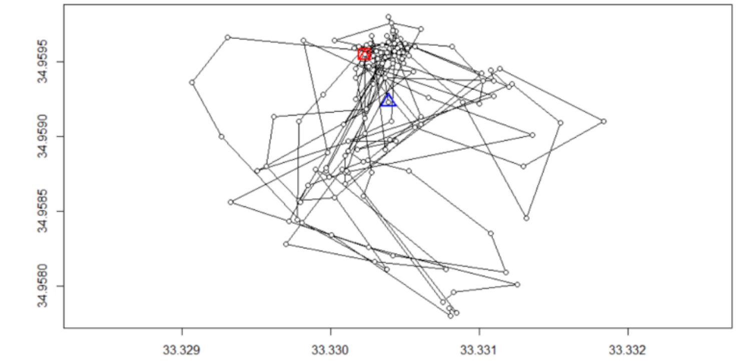

Trajectory

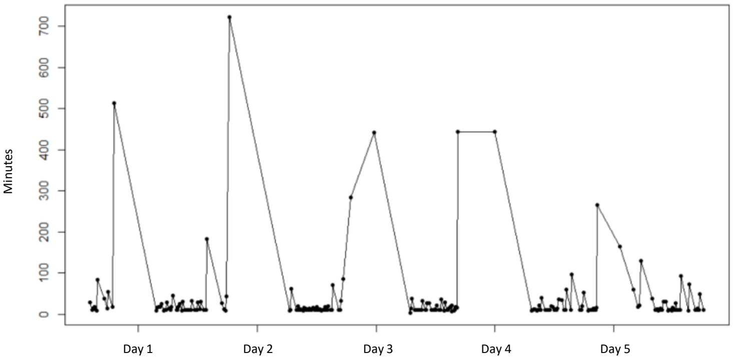

Time lag between GPS fixes

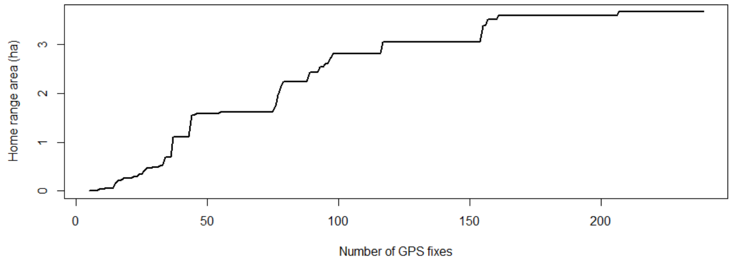

Cumulative MCP (100%) area by number of fixes

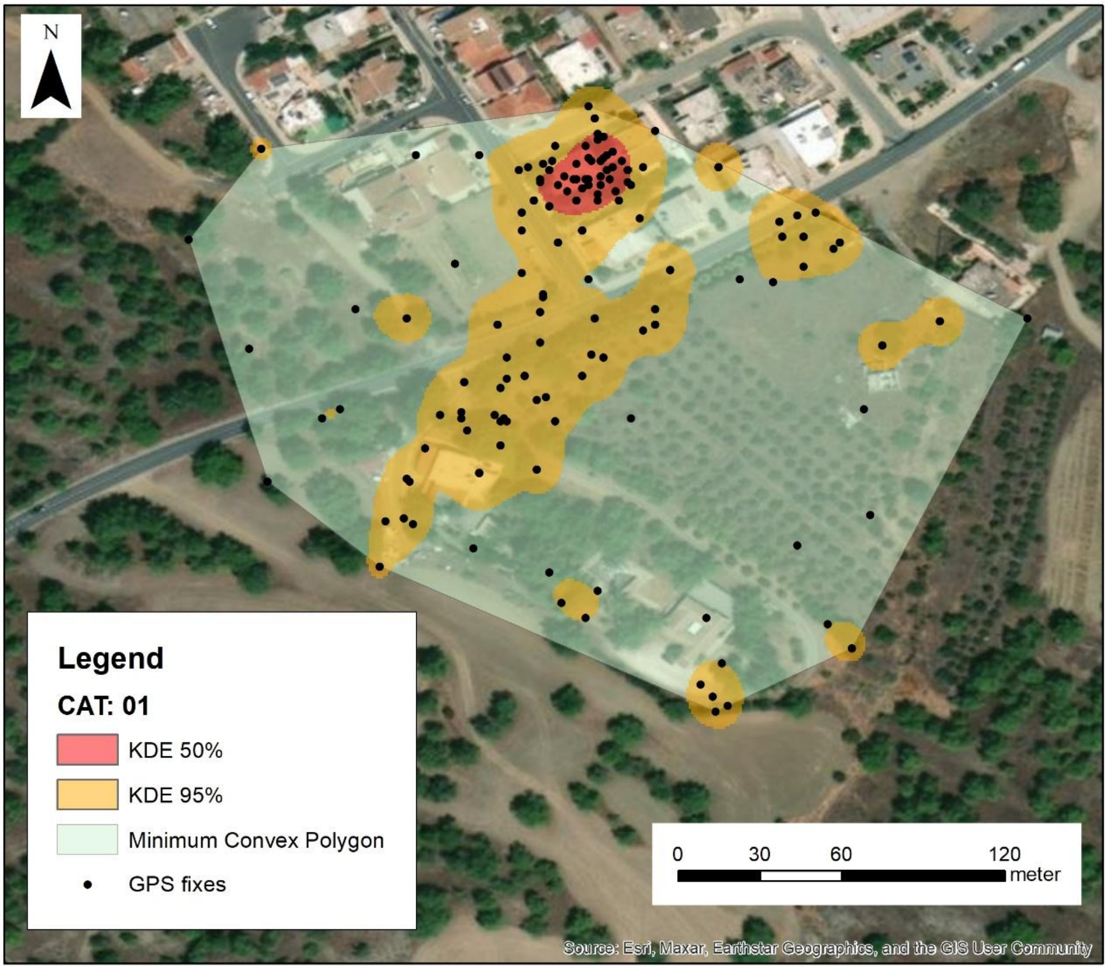

Home Range

CAT
ID 02

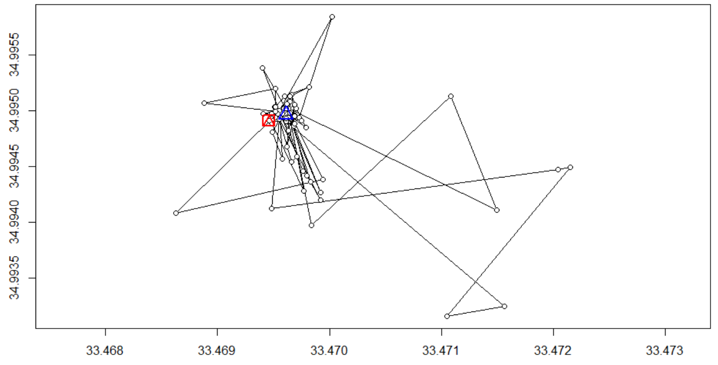

Trajectory

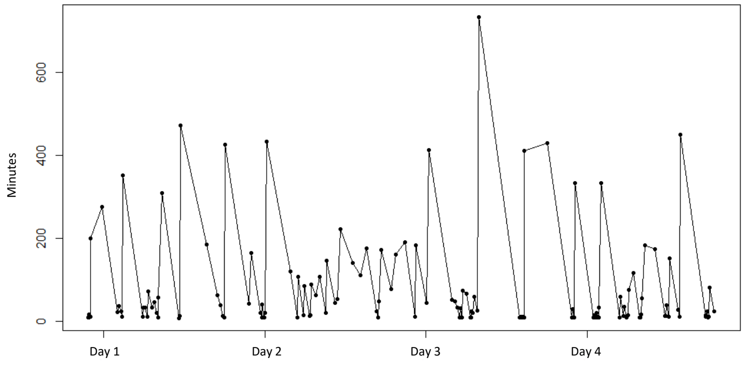

Time lag between GPS fixes

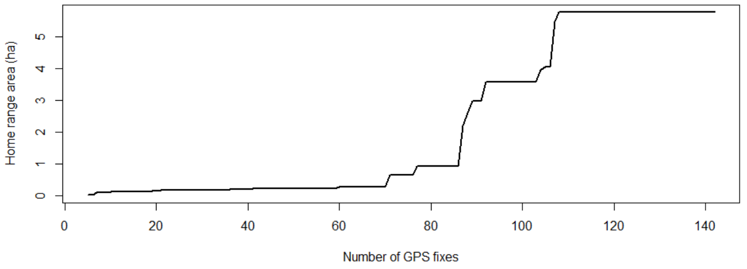

Cumulative MCP (100%) area by number of fixes

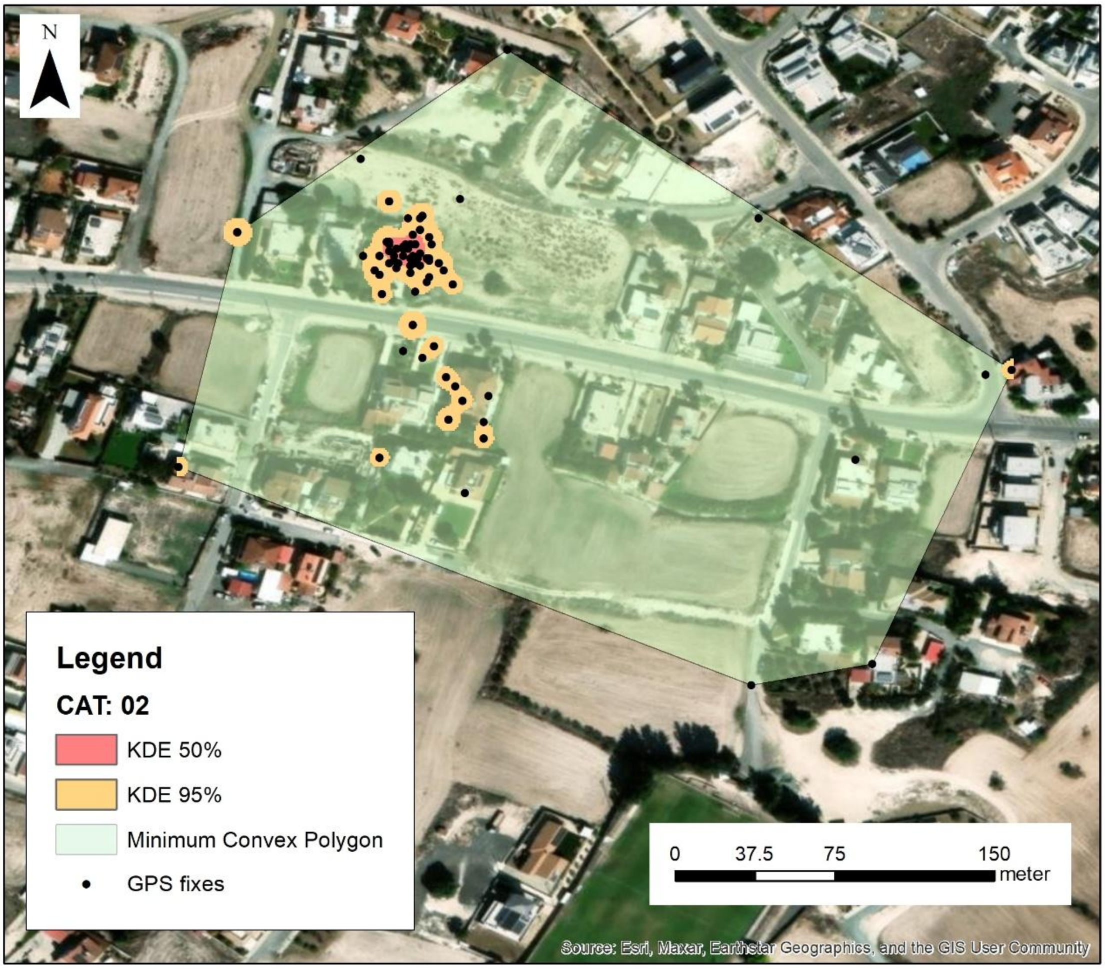

Home Range

CAT
ID 03

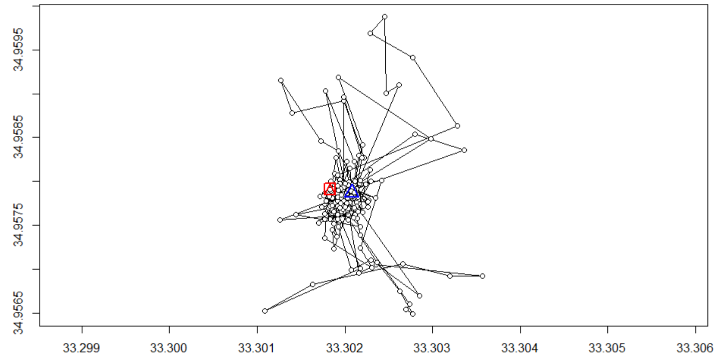

Trajectory

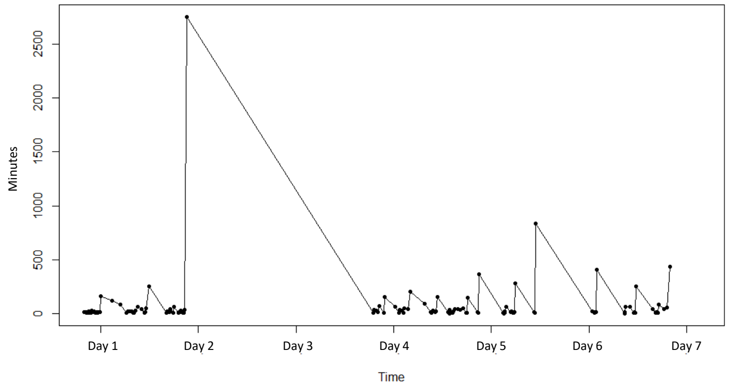

Time lag between GPS fixes

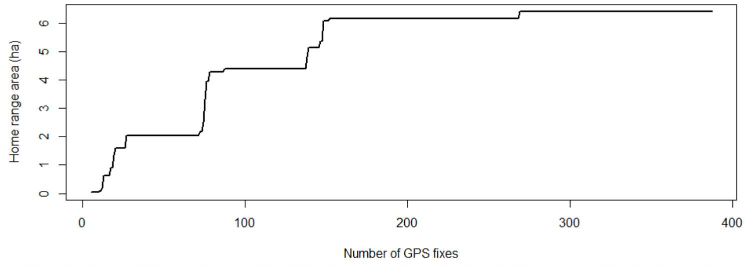

Cumulative MCP (100%) area by number of fixes

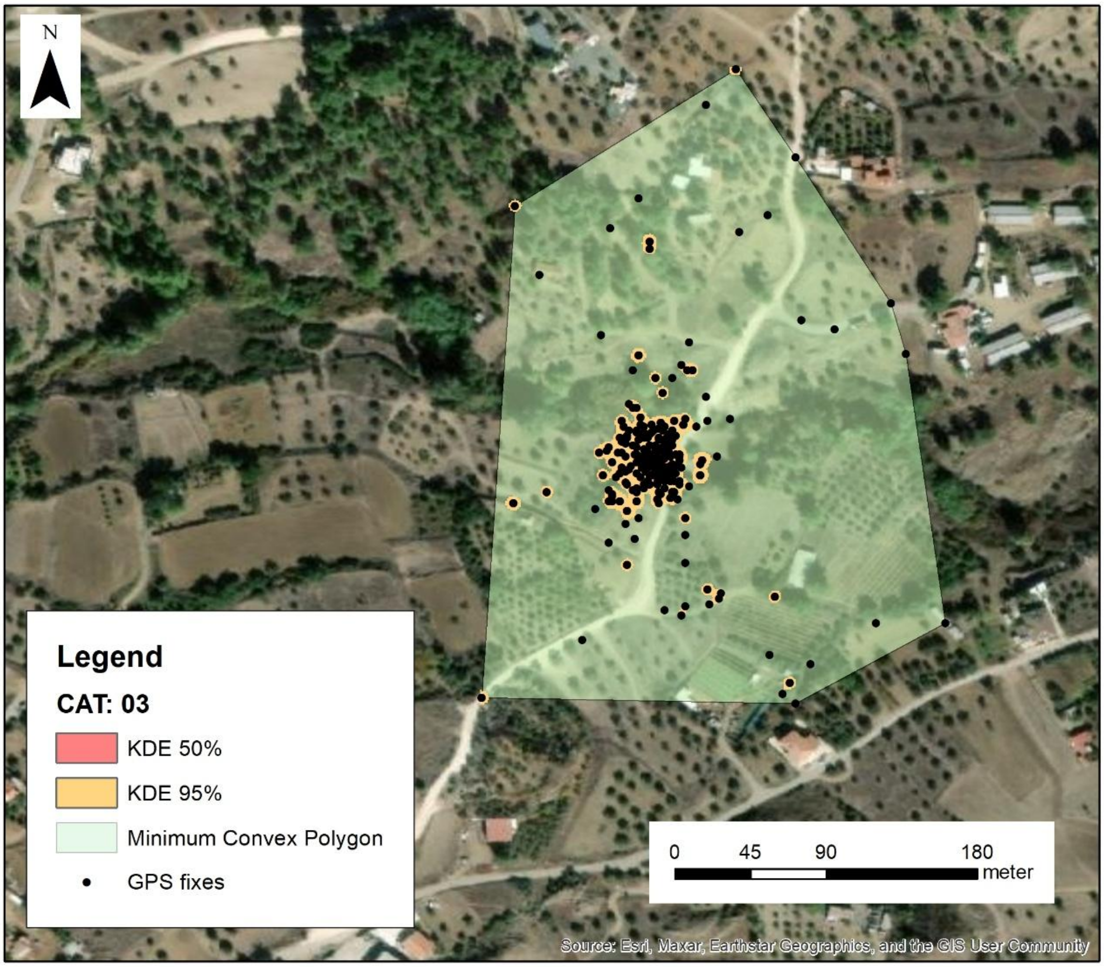

Home Range

CAT
ID 05

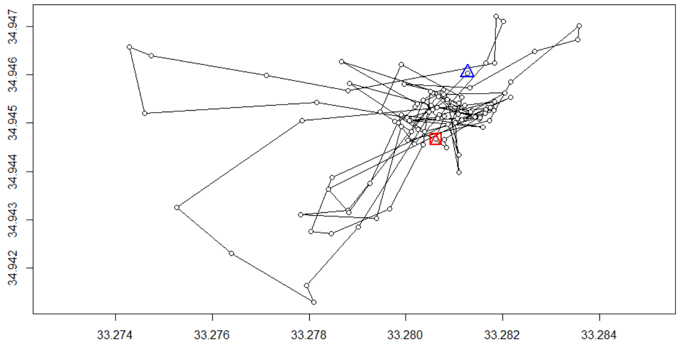

Trajectory

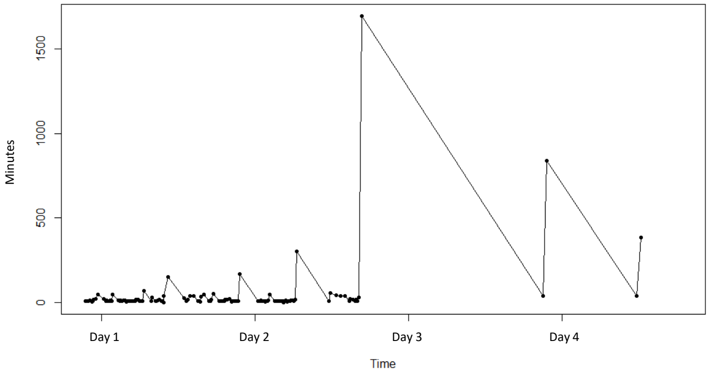

Time lag between GPS fixes

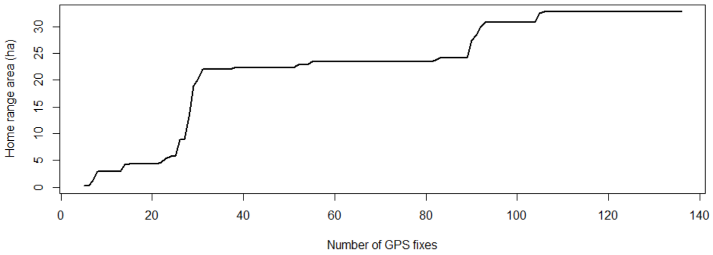

Cumulative MCP (100%) area by number of fixes

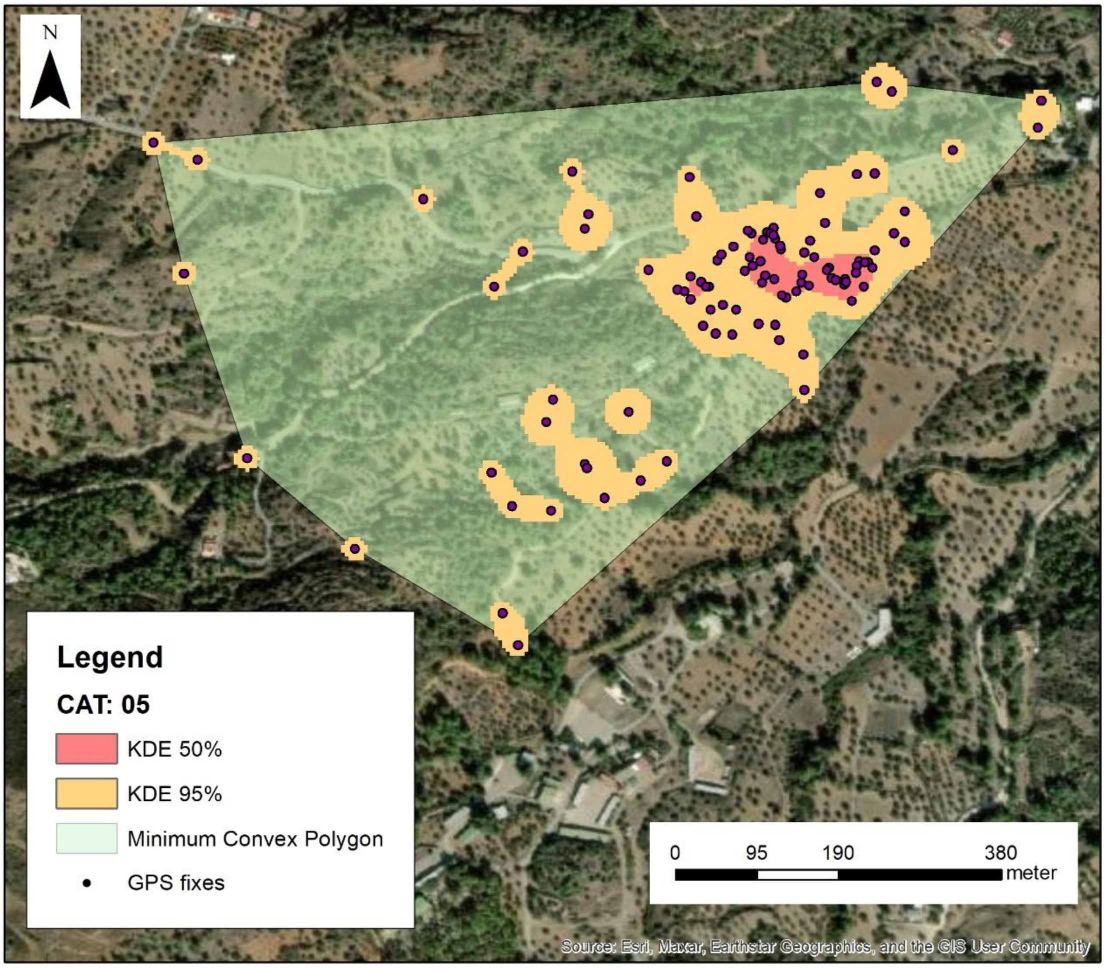

Home Range

CAT
ID 06

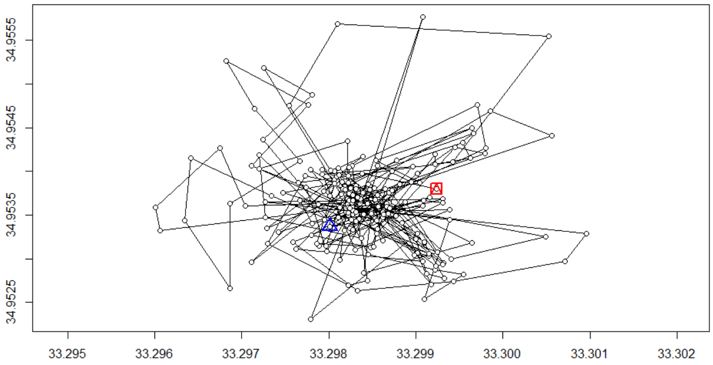

Trajectory

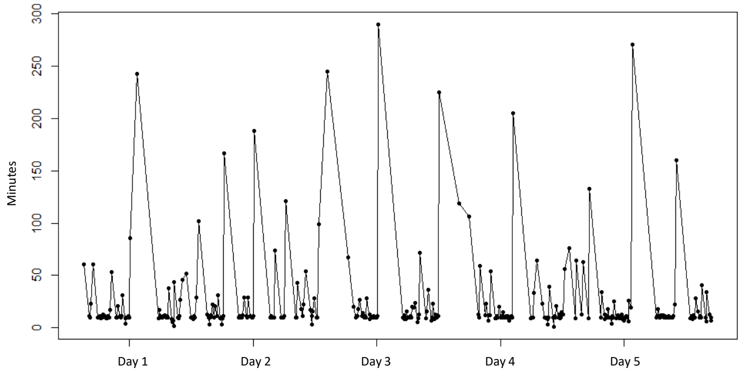

Time lag between GPS fixes

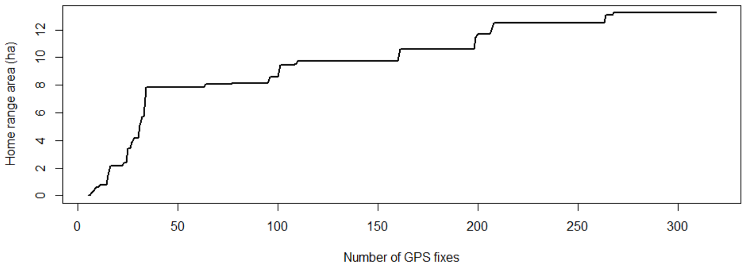

Cumulative MCP (100%) area by number of fixes

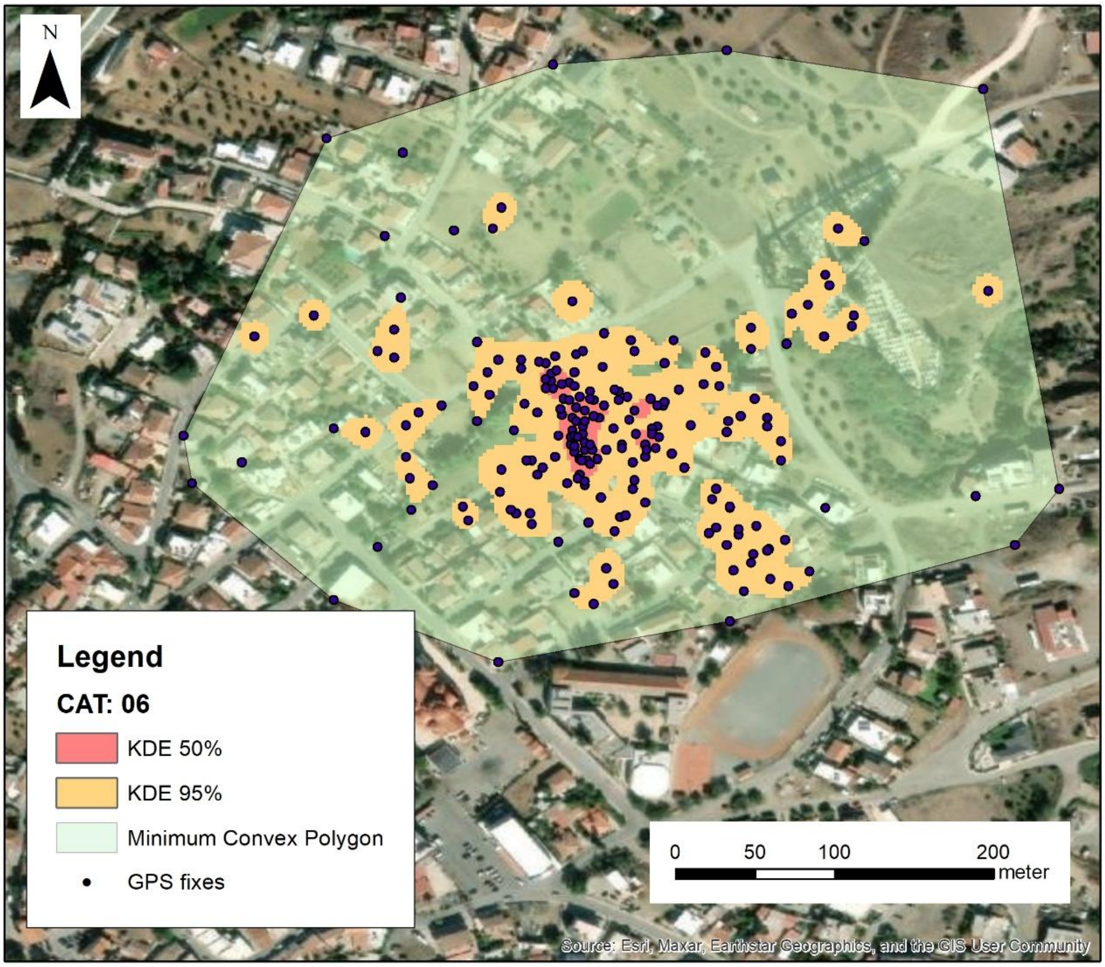

Home Range

CAT
ID 07

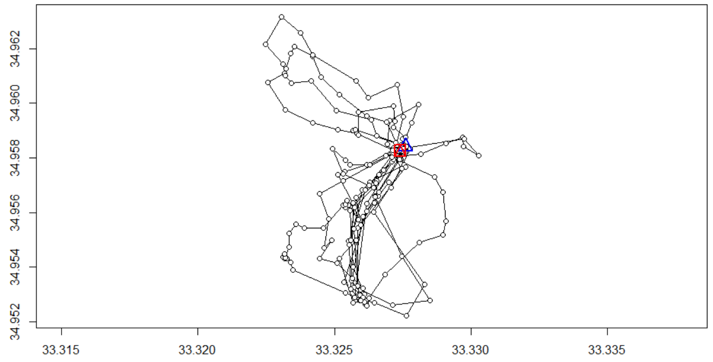

Trajectory

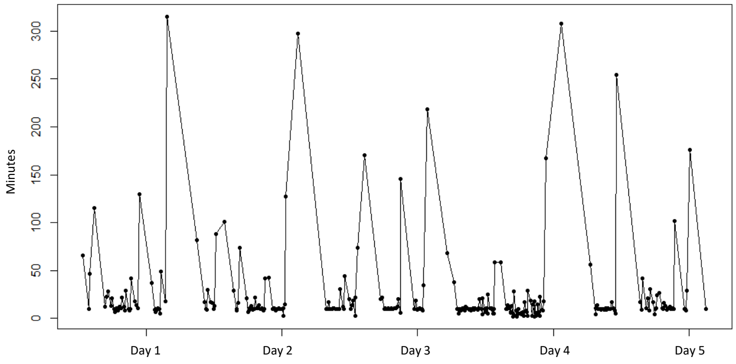

Time lag between GPS fixes

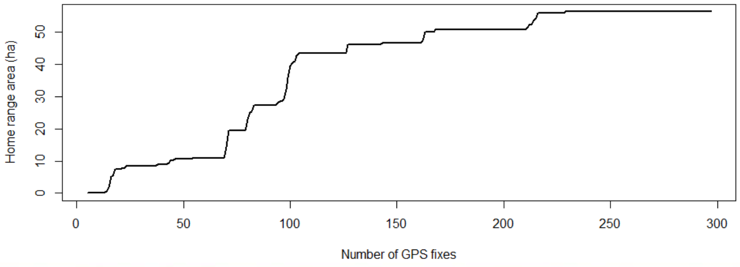

Cumulative MCP (100%) area by number of fixes

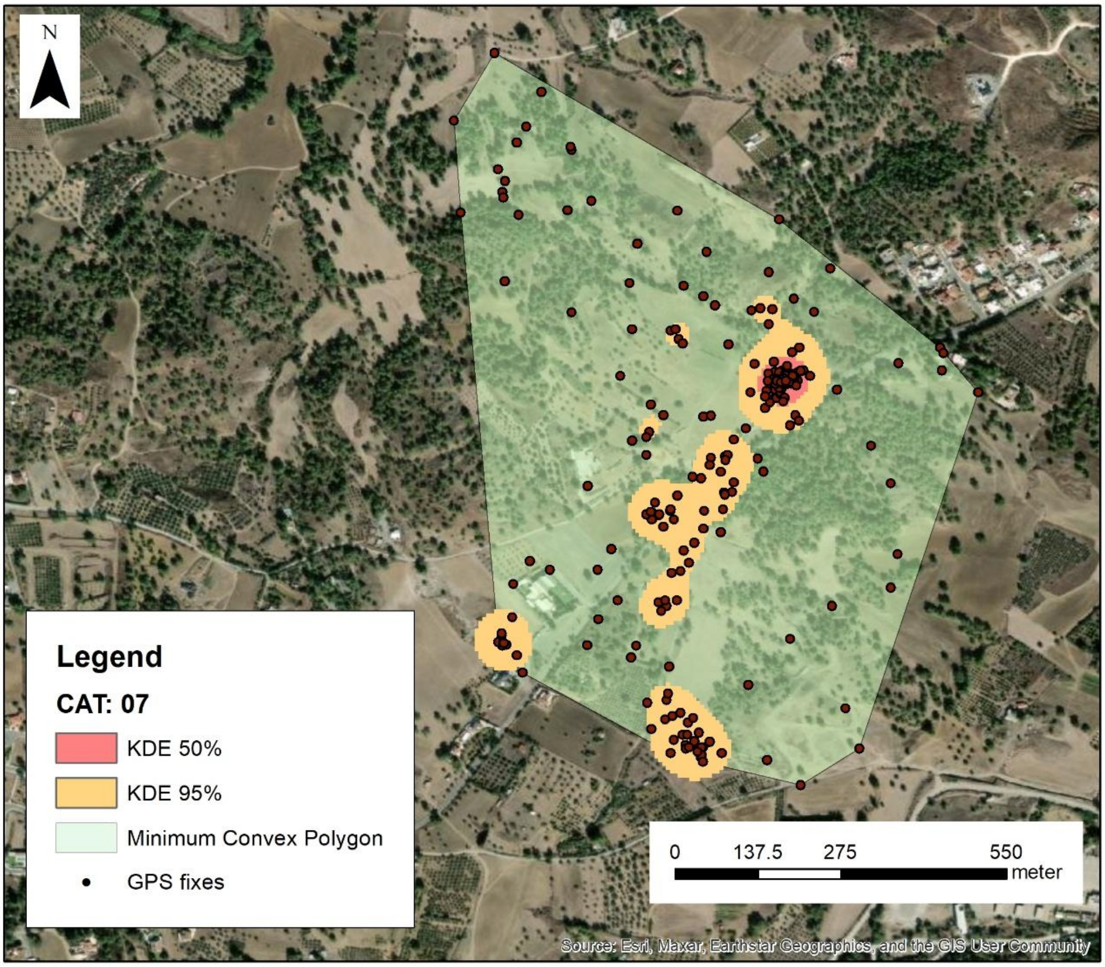

Home Range

CAT
ID 10

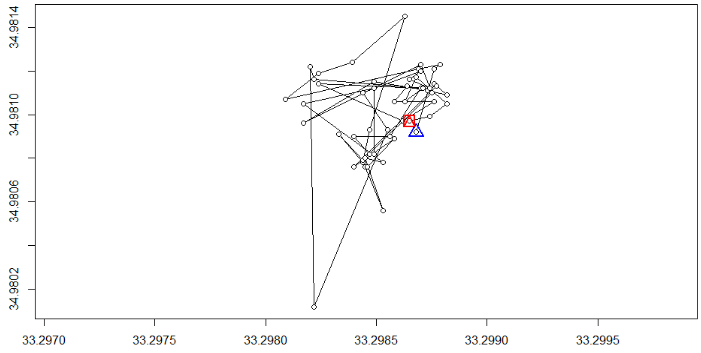

Trajectory

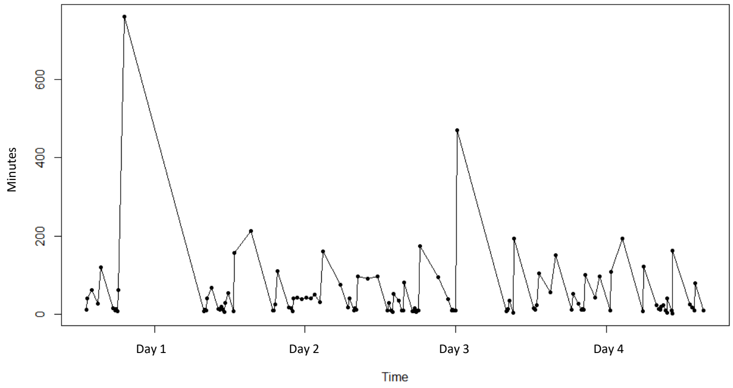

Time lag between GPS fixes

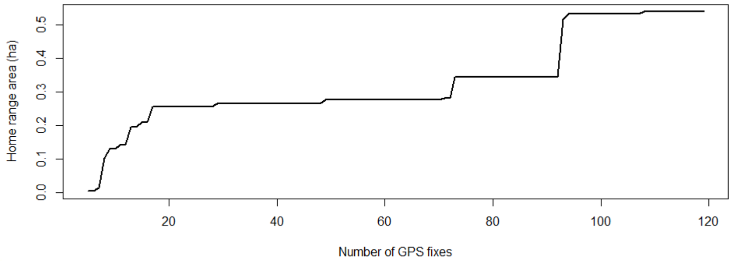

Cumulative MCP (100%) area by number of fixes

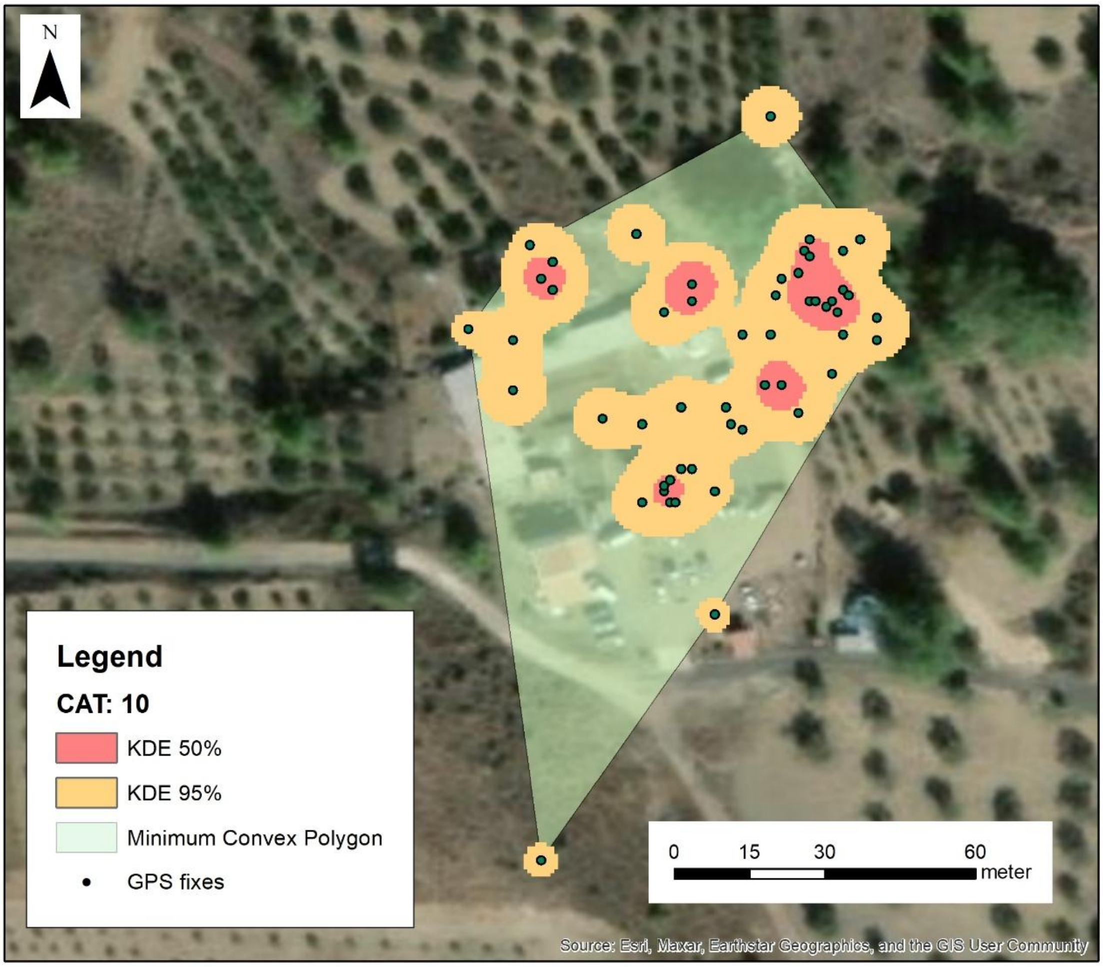

Home Range

CAT
ID 15

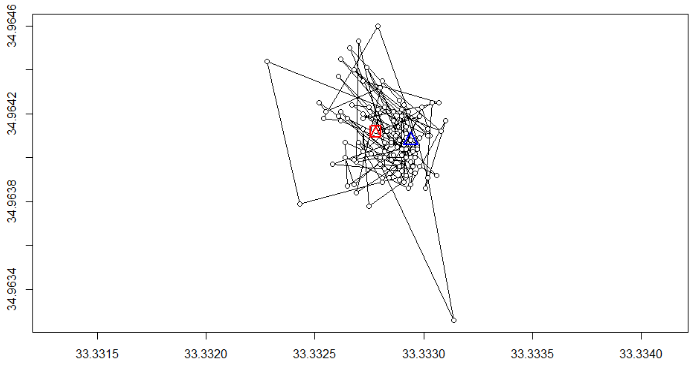

Trajectory

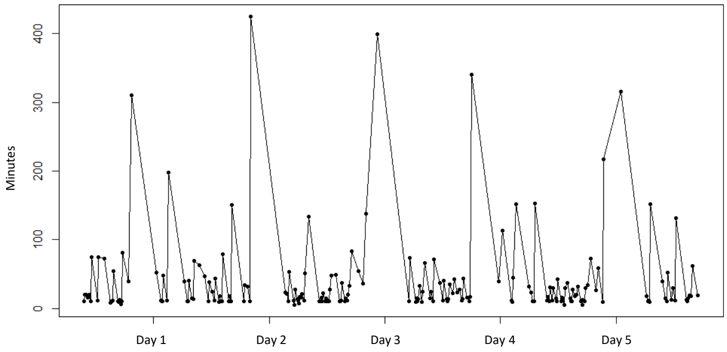

Time lag between GPS fixes

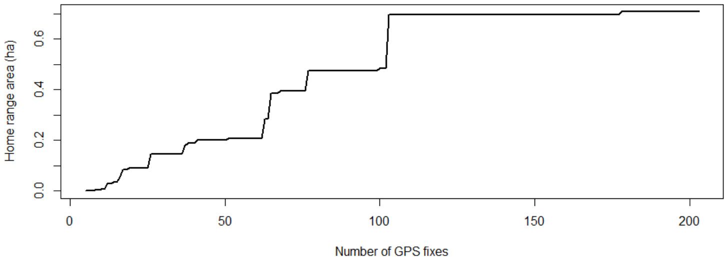

Cumulative MCP (100%) area by number of fixes

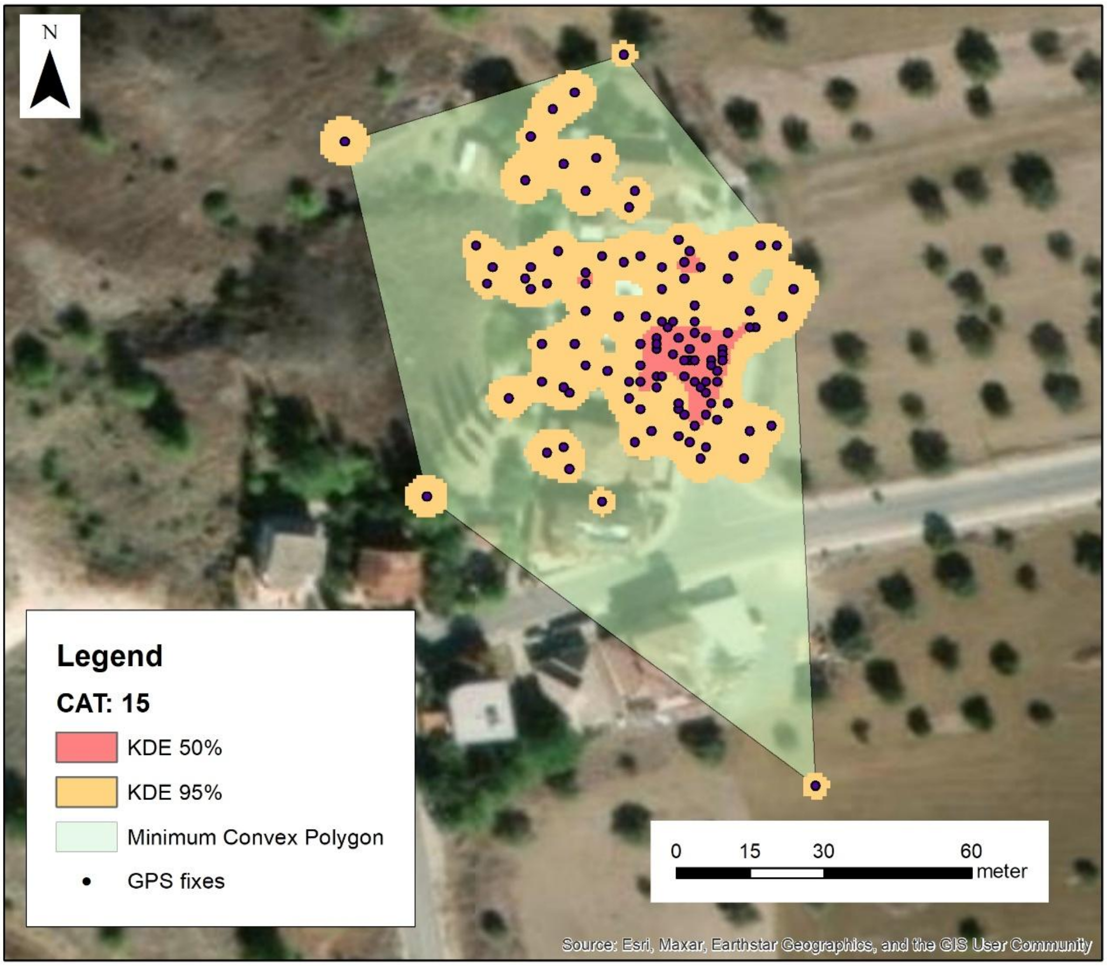

Home Range

CAT
ID 16

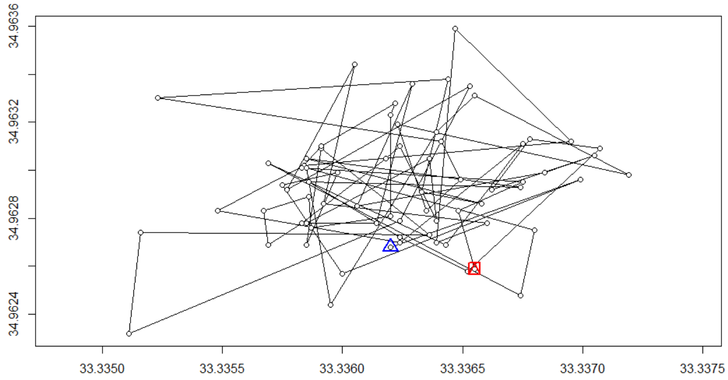

Trajectory

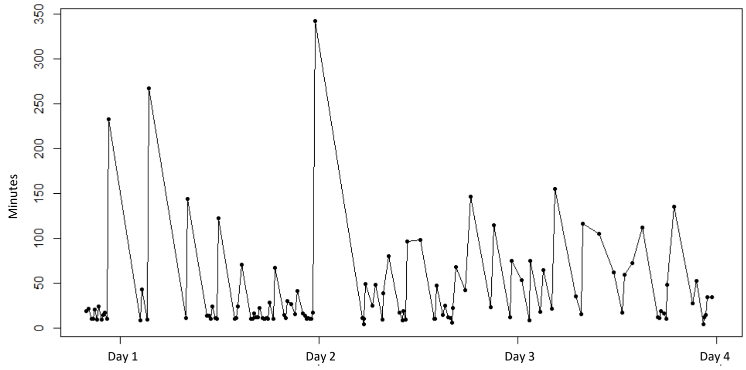

Time lag between GPS fixes

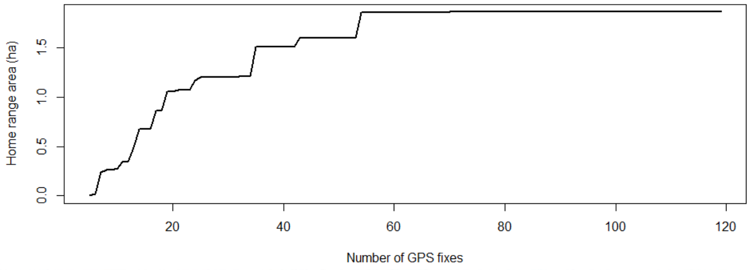

Cumulative MCP (100%) area by number of fixes

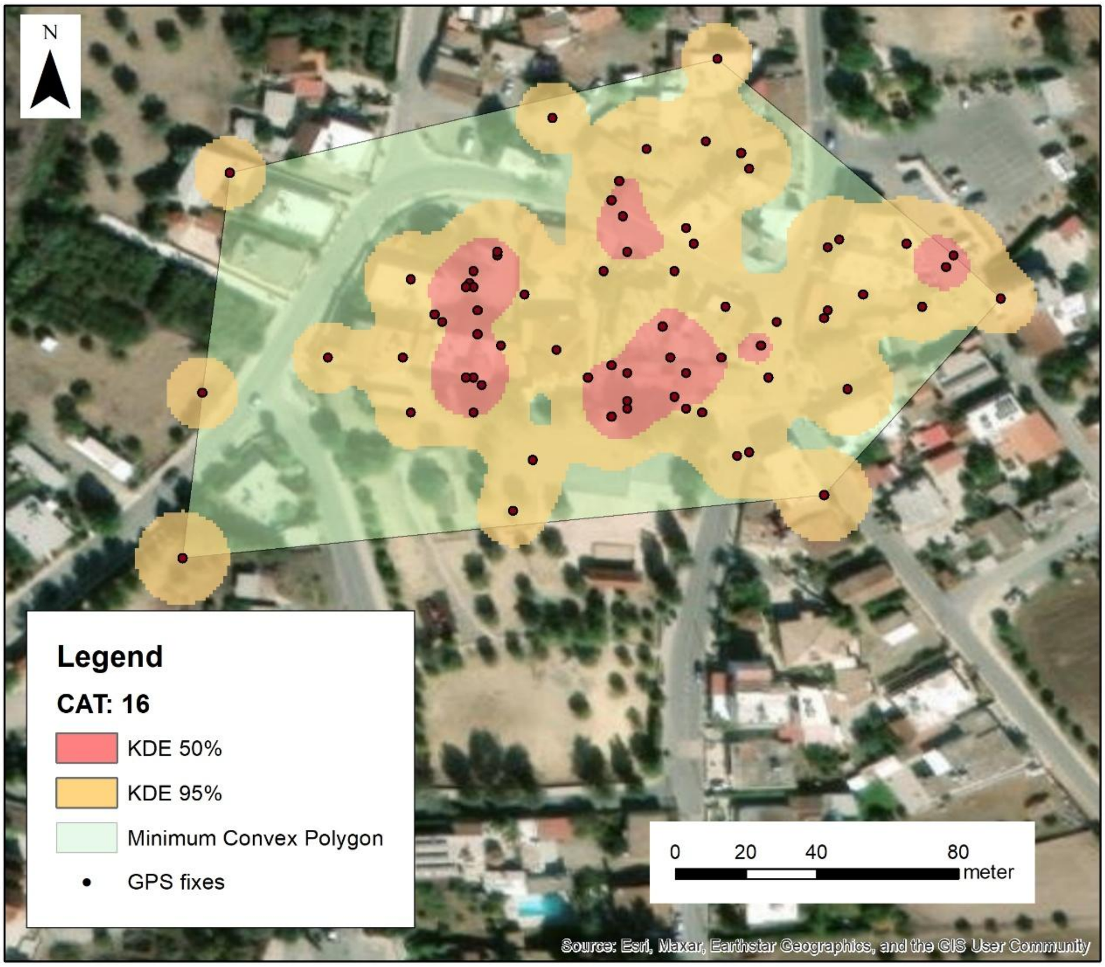

Home Range

CAT
ID 17

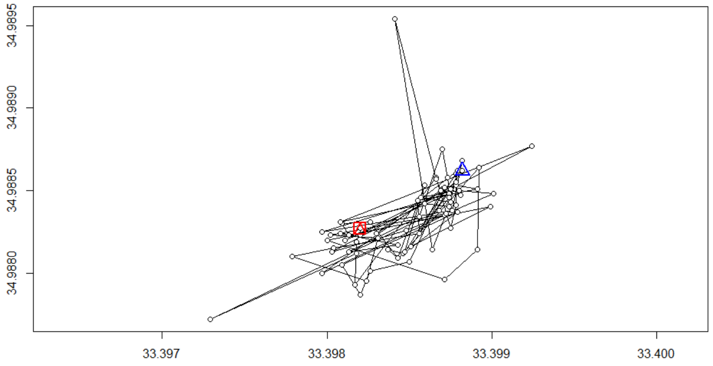

Trajectory

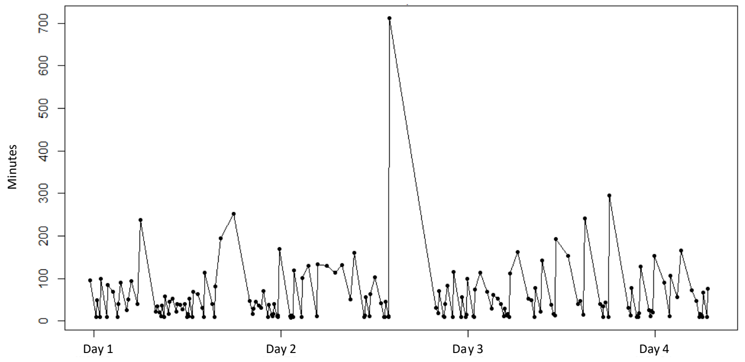

Time lag between GPS fixes

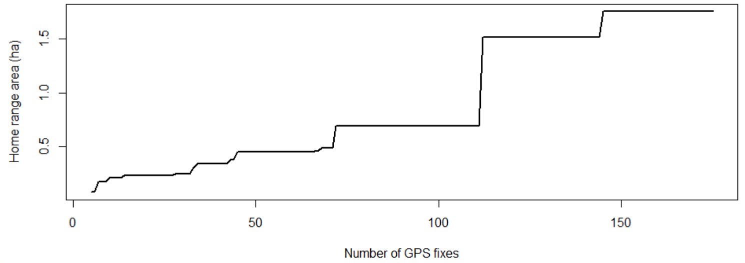

Cumulative MCP (100%) area by number of fixes

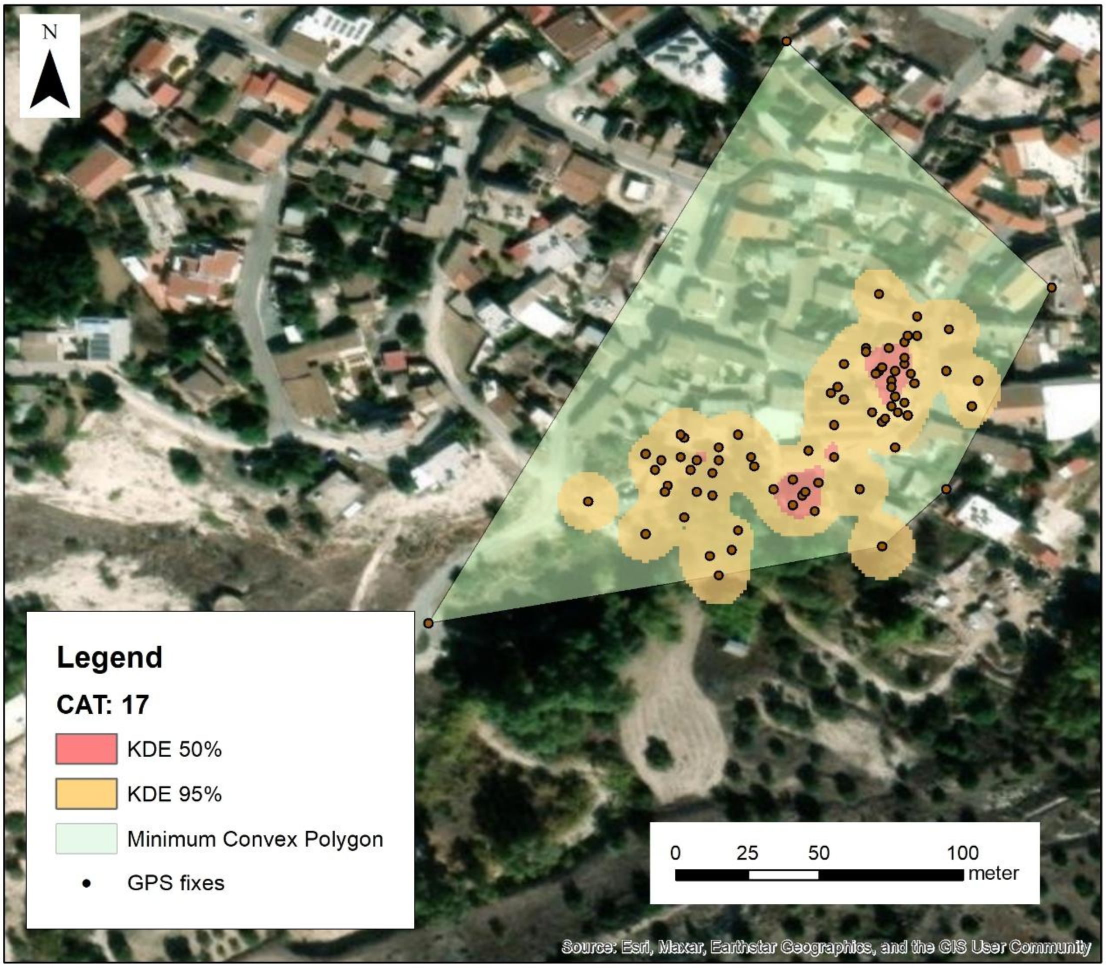

Home Range

CAT
ID 19

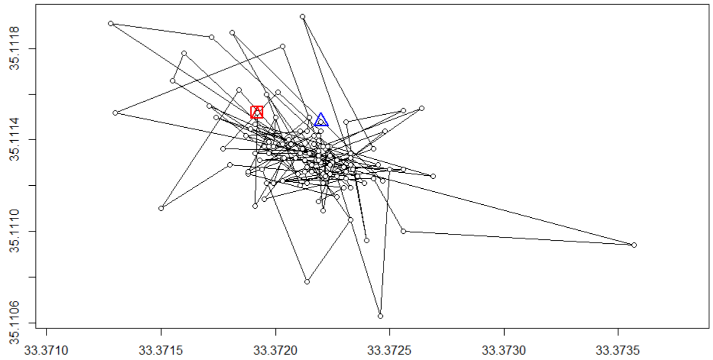

Trajectory

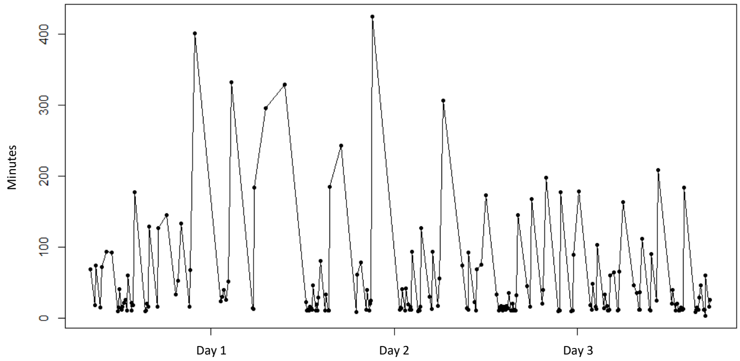

Time lag between GPS fixes

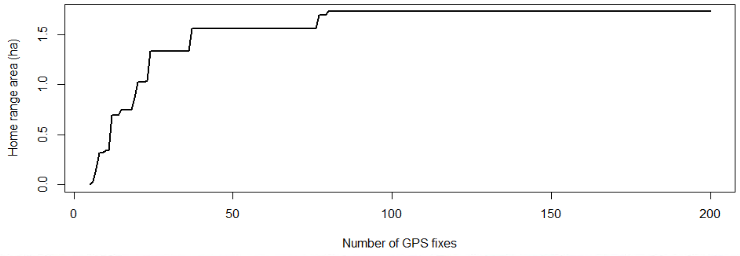

Cumulative MCP (100%) area by number of fixes

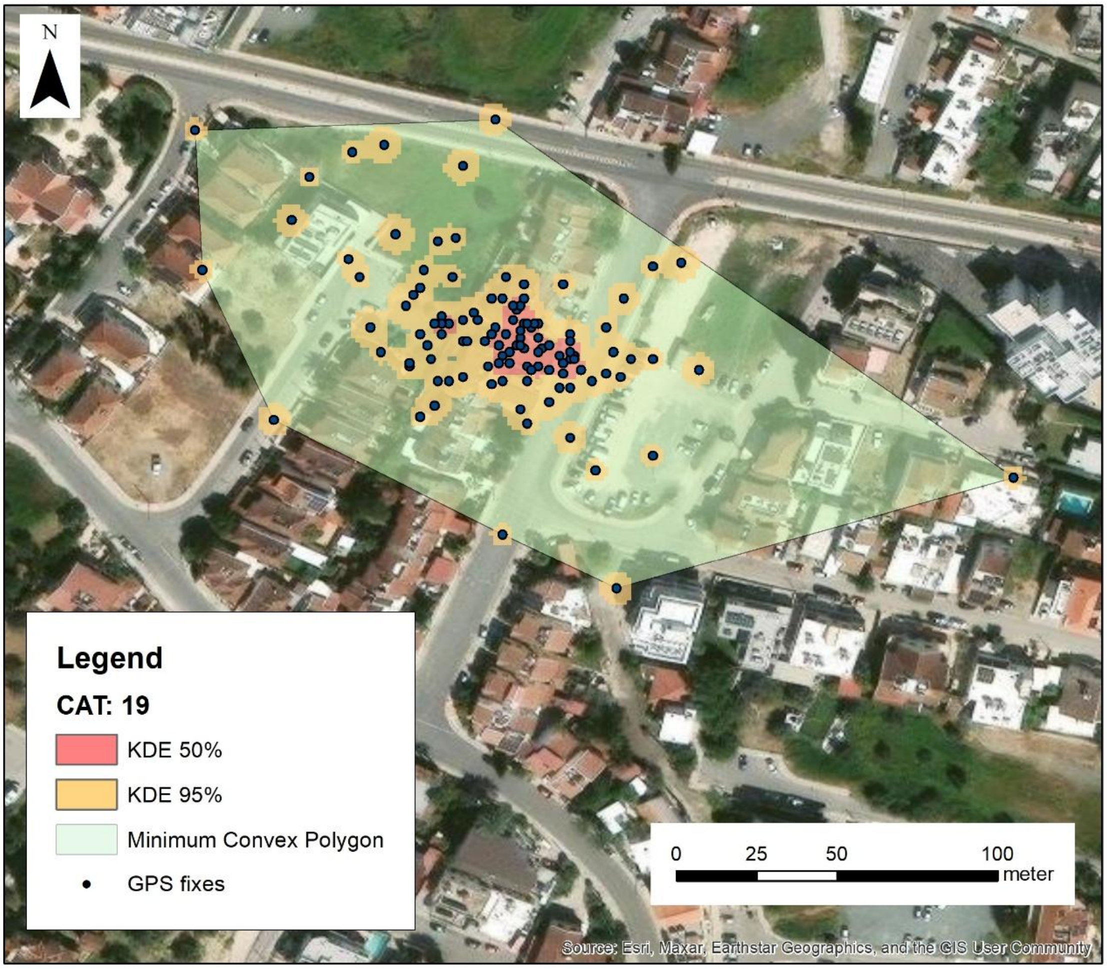

Home Range

CAT
ID 20

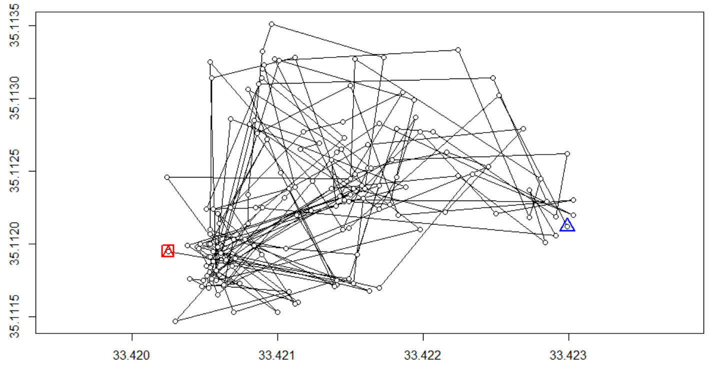

Trajectory

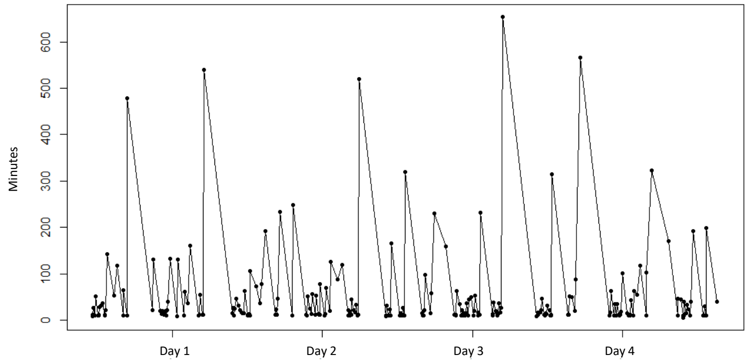

Time lag between GPS fixes

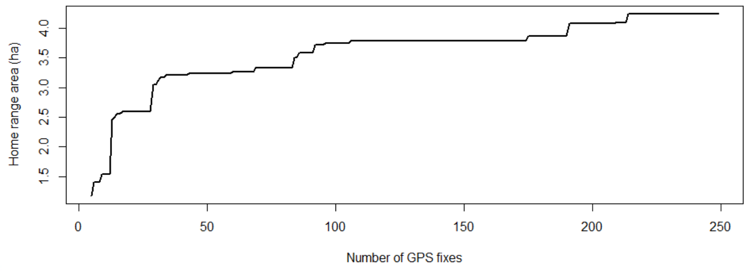

Cumulative MCP (100%) area by number of fixes

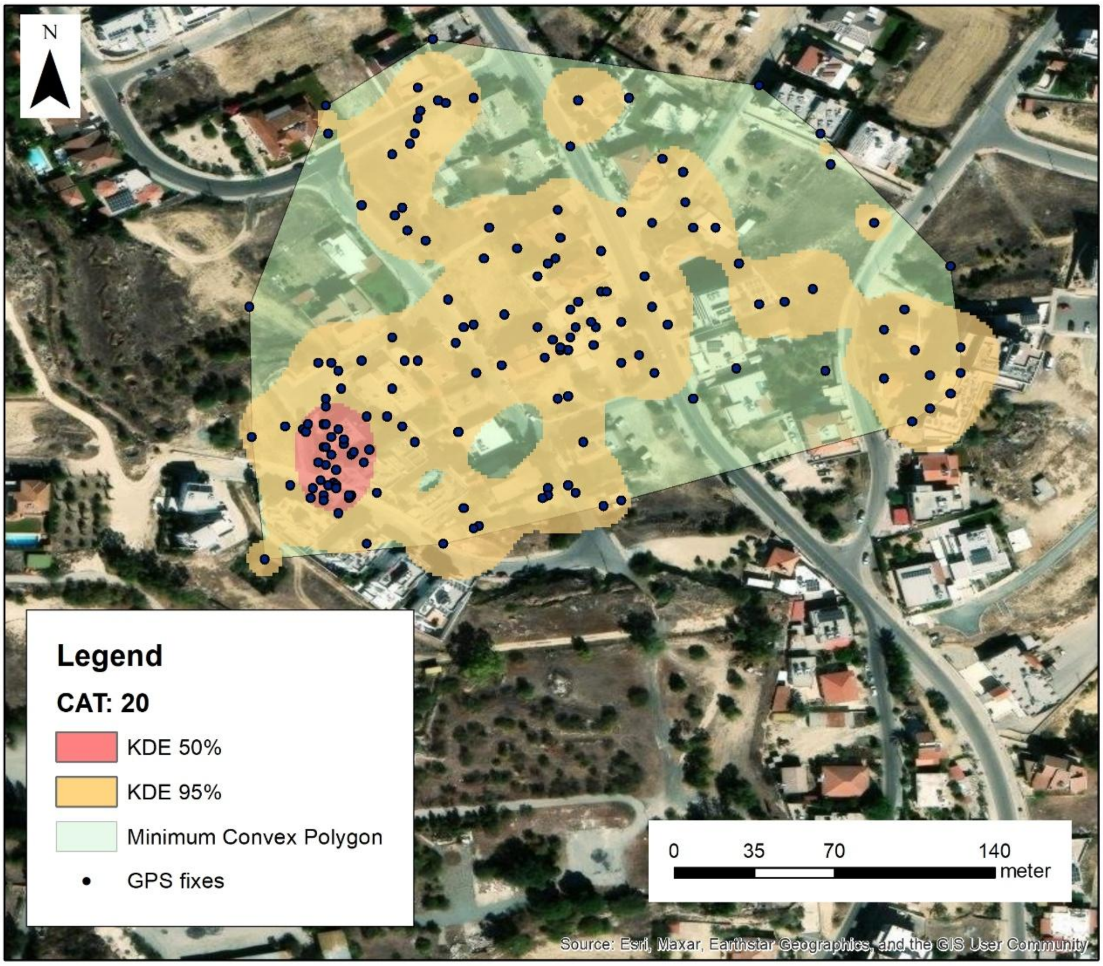

Home Range

CAT
ID 23

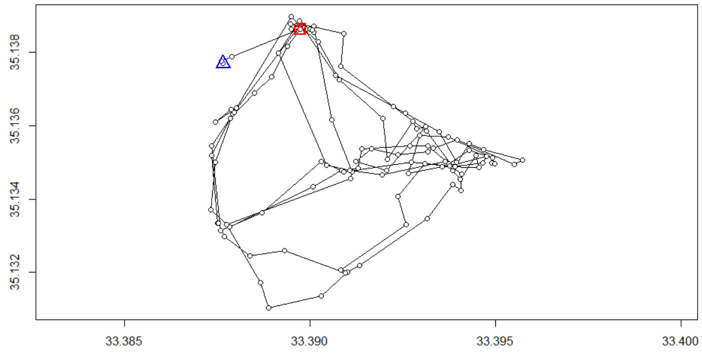

Trajectory

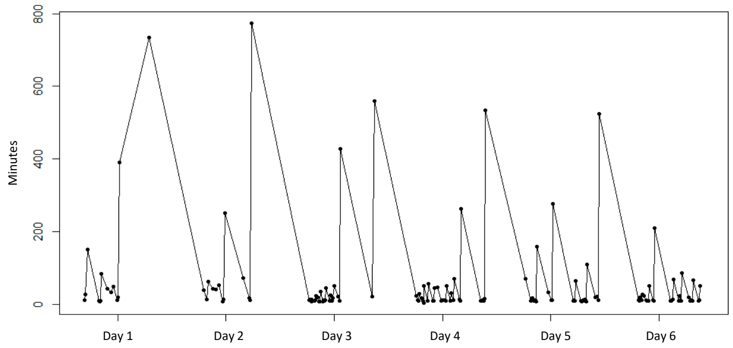

Time lag between GPS fixes

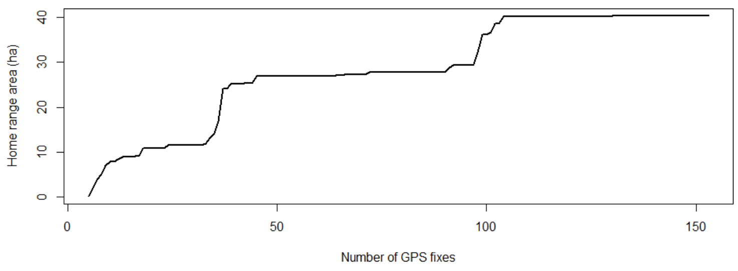

Cumulative MCP (100%) area by number of fixes

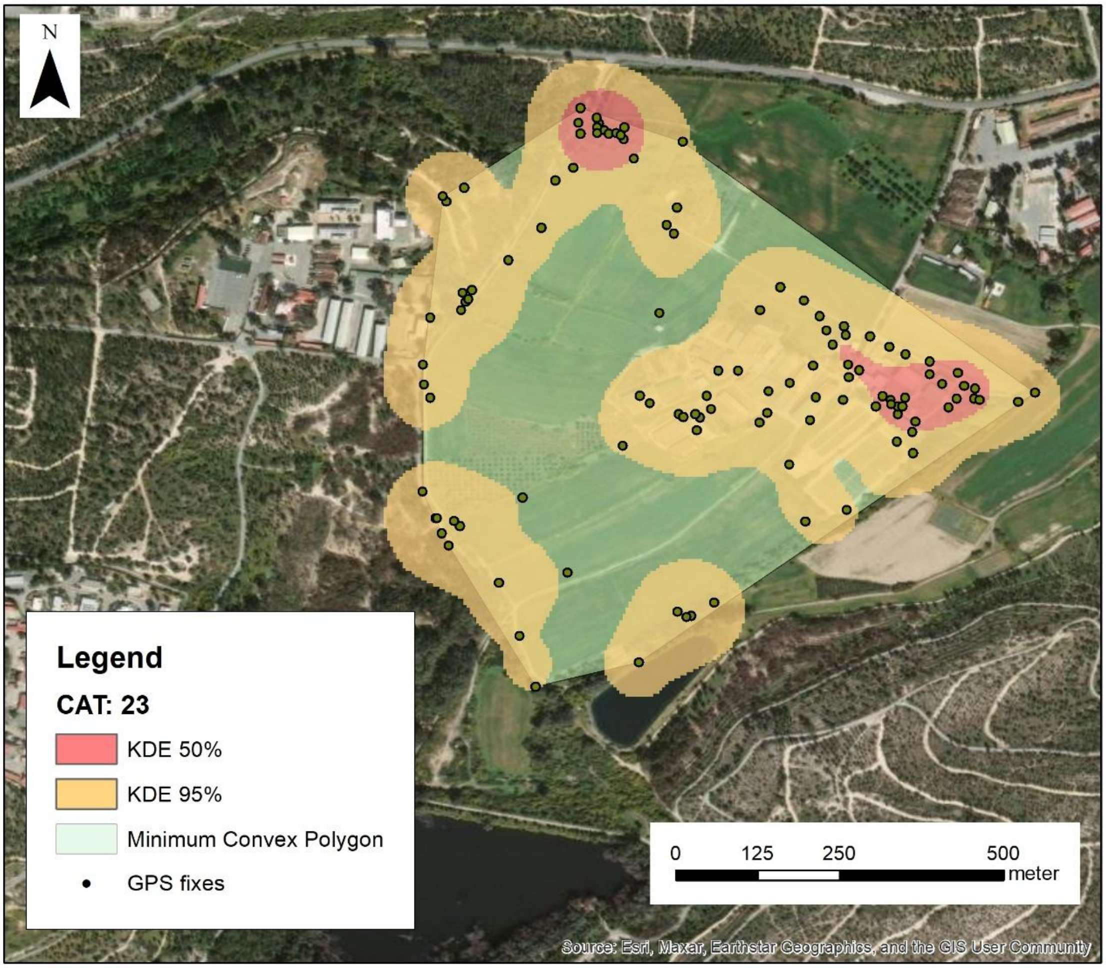

Home Range

CAT
ID 24

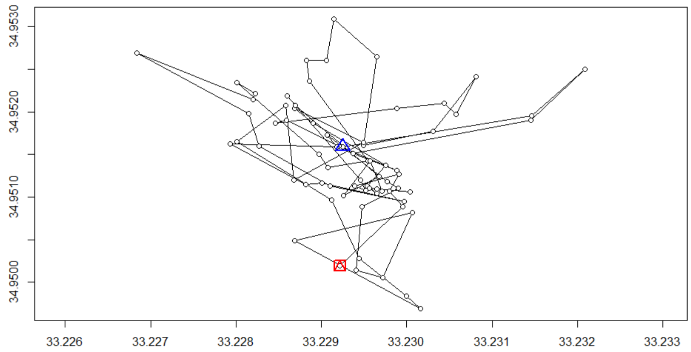

Trajectory

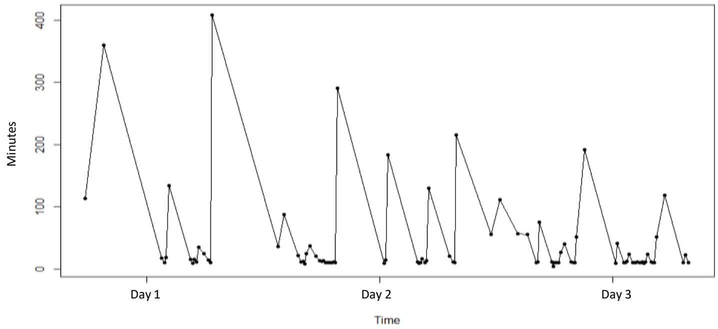

Time lag between GPS fixes

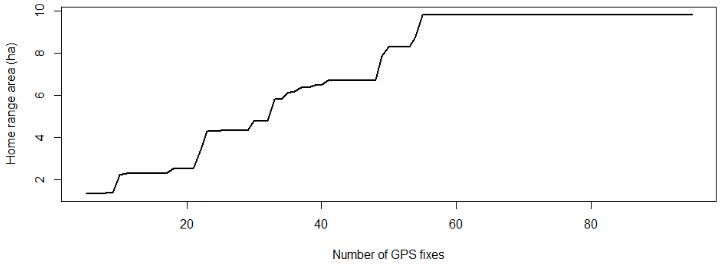

Cumulative MCP (100%) area by number of fixes

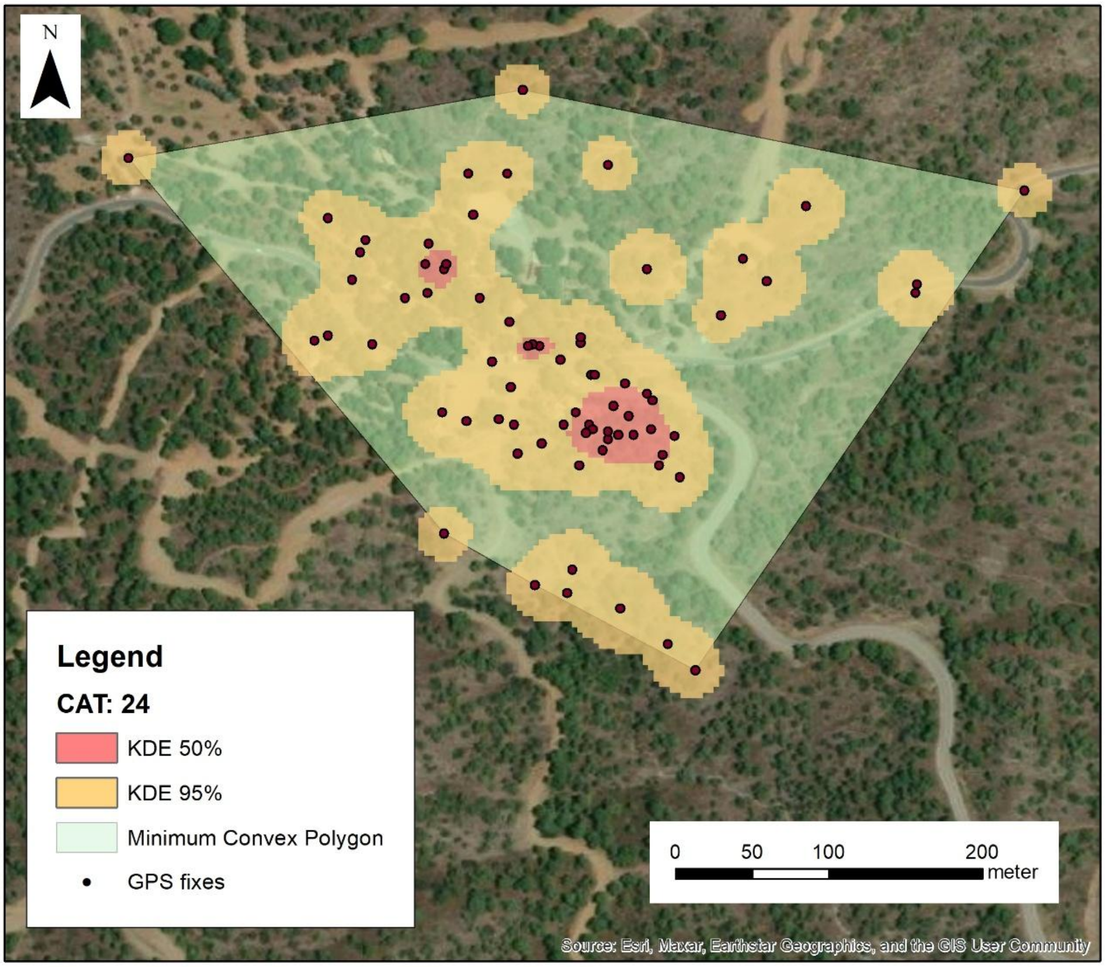

Home Range

CAT
ID 26

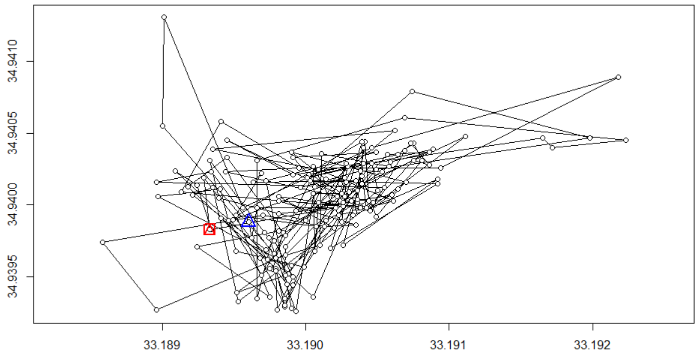

Trajectory

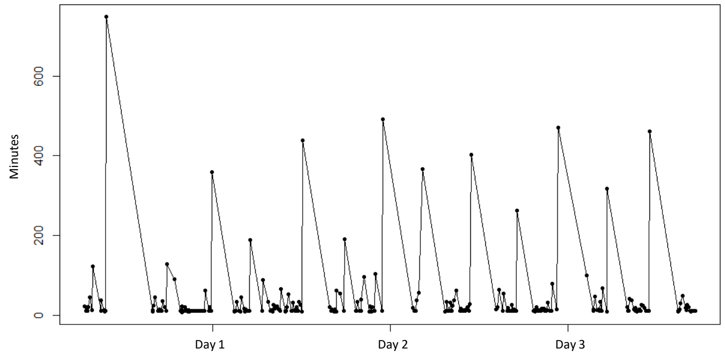

Time lag between GPS fixes

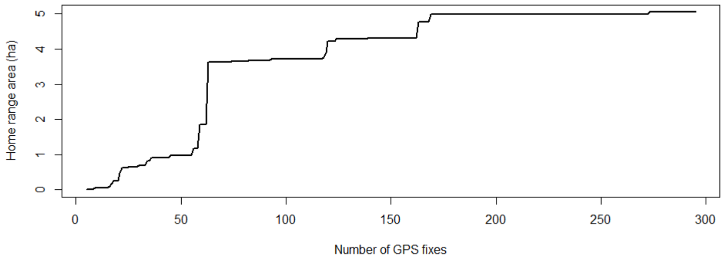

Cumulative MCP (100%) area by number of fixes

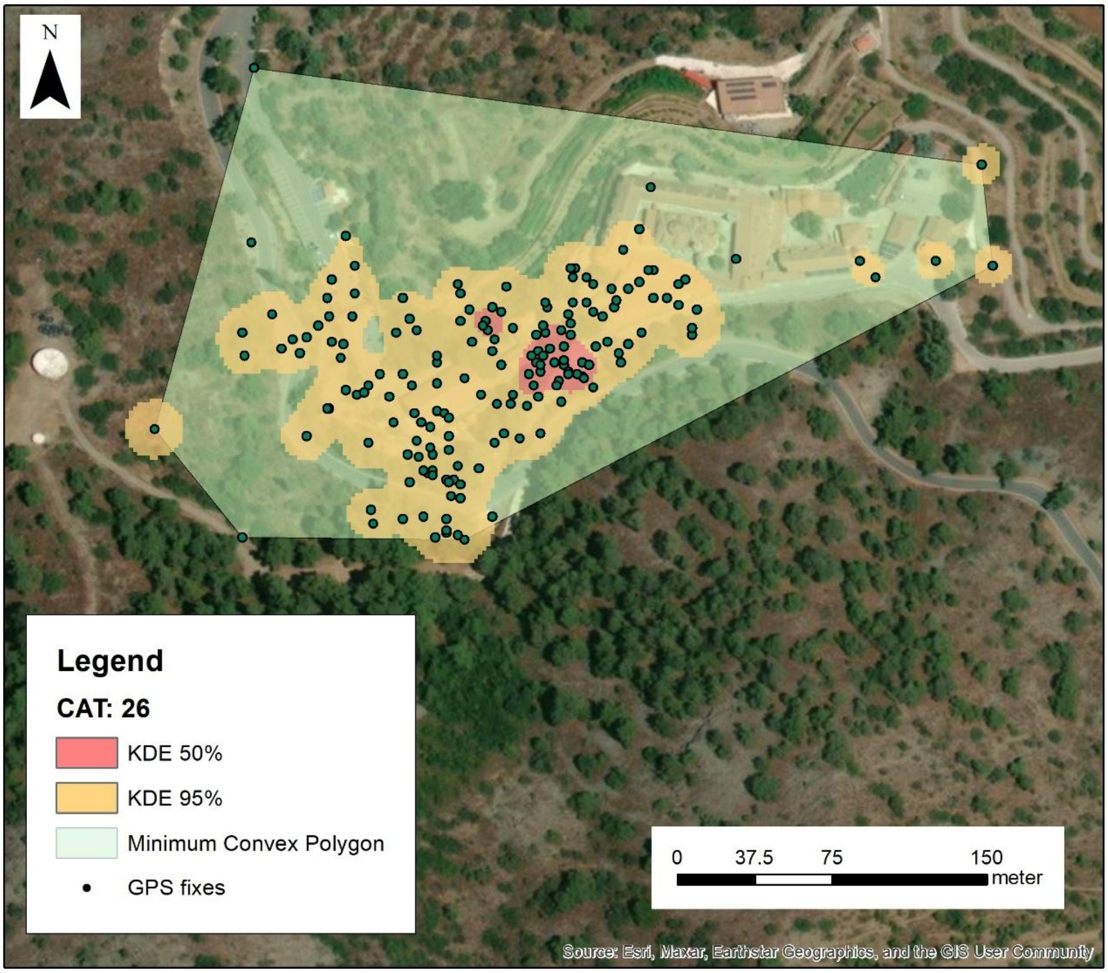

Home Range
