# Supplementary material for: Living Wild in a Mediterranean Island: Spatial and Temporal Behaviour of Free-Roaming Cats in Cyprus
Source: Animals (Basel). 2026 Apr 3;16(7):1101. doi: 10.3390/ani16071101 (PMC13072416; doi:10.3390/ani16071101)
Supplement: Supplementary file 1 [file animals-16-01101-s001.zip › Suppementary File S1.pdf]

Living Wild in a Mediterranean Island: Spatial and Temporal Behaviour of Free-Roaming Cats in Cyprus

Michalis Zacharia, Ioannis N. Vogiatzakis and Savvas Zotos

Supplementary file S1

Individual data from the 15 monitoring cats, accompanied by data on monitoring start date/time, monitoring duration, Home Range, percentage of overlapping area and distance travelled in meters.

Table S1.1: Individual data from the 15 monitoring cats, accompanied by data on the monitoring start date/time and duration, Home Range (KDE95 - Kernel Density Estimation 95%; MCP100 - Minimum Convex Polygon 100%) Home Range Core area (KDE50 - Kernel Density Estimation 50%), and percentage of Forested (%Forest), Agricultural (%Agri) and Urban (%Urban) area within the MCP100.

Individual data					Monitoring data			Home Range (m2)			% of area used within MCP100		
ID	Sex	Age_class	Weigth	Shelter	Start date/time	duration (h)	Fixes	KDE50	KDE95	MCP100	% Forest	% Agri	% Urban
ID01	F	Mature	2.5	Yes	12/03/2022 14:17	124	239	6,826	40,180	36,689	26%	42%	32%
ID02	M	Mature	3.5	Yes	12/03/2022 19:26	186	142	2,961	31,166	57,542	0%	38%	62%
ID03	M	Young	2.5	Yes	04/04/2022 19:31	218	386	2,059	28,401	63,950	11%	85%	4%
ID05	M	Mature	3	No	08/04/2022 21:26	93	136	39,611	325,011	327,116	72%	26%	1%
ID06	M	Mature	3	No	07/04/2022 15:10	122	320	10,888	86,577	132,186	27%	19%	54%
ID07	M	Young	3	Yes	09/04/2022 12:43	110	297	127,072	655,373	563,140	66%	33%	1%
ID10	F	Mature	3	Yes	16/04/2022 13:10	98	119	1,416	5,967	5,395	15%	35%	49%
ID15	F	Mature	2.5	Yes	07/05/2022 09:33	127	203	1,050	5,021	7,078	19%	22%	59%
ID16	M	Young	3.5	No	15/05/2022 19:50	76	119	5,131	21,089	18,602	0%	10%	90%
ID17	F	Mature	3	Yes	25/05/2022 23:00	159	175	2,992	11,594	17,550	17%	0%	83%
ID19	F	Mature	3	Yes	26/05/2022 16:32	162	200	1,399	11,160	17,283	0%	16%	84%
ID20	M	Mature	4	Yes	02/08/2022 22:24	200	249	13,425	55,678	42,447	0%	35%	65%
ID23*	M	Mature	4.5	No	27/07/2022 16:39	137	153	191,391	849,732	403,015	20%	69%	10%
ID24*	F	Young	3	No	23/09/2022 16:30	63	95	23,406	114,033	98,364	99%	0%	1%
ID26*	M	Young	5	No	21/10/2022 13:29	165	295	7,886	36,088	50,440	58%	7%	34%

* captured with Tomahawk traps

Table S1.2: Individual data from the 15 monitoring cats, accompanied by data on the distance travelled in meters. Max_L_dist: Maximum linear distance travelled in meters from the feeding station; Daily - 24h: daily distance travelled in meters (24 h; midnight to midnight); Morning (until dawn):
Daytime (dawn - dusk): distance travelled in meters during daytime (dawn to dusk); Evening (after dusk): distance travelled in meters during evening hours (dusk until the last activity of the day);
SD: Standard Deviation

Individual data					Distance travelled								
						Daily - 24h		Morning (until dawn)		Daytime (dawn - dusk)		Evening (after dusk)	
ID	Sex	Age_class	Weigth	Shelter	max_L_ dist	Average	(min - max) SD	Average	(min - max) SD	Average	(min - max) SD	Average	(min - max) SD
ID01	F	Mature	2.5	Yes	266	1,030	(955 - 1,501) 392	98	(22 -295) 193	895	(901 - 1,389) 345	53	(0 - 232) 164
ID02	M	Mature	3.5	Yes	202	404	(117 - 1,125) 713	272	(0 - 947) 670	104	(0 - 248) 175	78	(0 - 212) 150
ID03	M	Young	2.5	Yes	254	1,002	(374 - 1,347) 688	377	(187 - 847) 466	466	(0 - 781) 552	303	(28 - 1,078) 742
ID05	M	Mature	3	No	613	3,714	(4,361 - 5,801) 1,018	3,411	(3078 - 3745) 472	1,137	(561 - 1,713) 814	681	(980 - 1,010) 22
ID06	M	Mature	3	No	308	2,778	(2,310 - 3,949) 1,158	1,016	(589 - 1731) 808	947	(541 - 1682) 807	1,182	(672 - 1,998) 938
ID07	M	Young	3	Yes	673	3,033	(3,672 - 4,878) 852	459	(116 - 969) 410	1,892	(1,230 - 2,842) 1,140	1,288	(460 - 1,880) 1,004
ID10	F	Mature	3	Yes	121	263	(295 - 384) 63	11	(0 - 45) 32	160	(138 - 263) 89	118	(0 - 245) 173
ID15	F	Mature	2.5	Yes	84	492	(415 - 824) 289	179	(110 - 3518) 170	321	(294 - 495) 142	26	(8 - 73) 46
ID16	M	Young	3.5	No	164	944	(423 - 2,026) 1,134	137	(89 - 230) 100	760	(183 - 1443) 891	271	(84 - 494) 290
ID17	F	Mature	3	Yes	164	487	(353 - 710) 253	230	(73 - 465) 277	157	(39 - 469) 304	117	(66 - 201) 98
ID19	F	Mature	3	Yes	114	583	(190 - 1,194) 710	287	(38 - 557) 367	301	(49 - 1,002) 674	36	(0 - 61) 43
ID20	M	Mature	4	Yes	104	505	(967 - 1,749) 552	126	(190- 609) 296	241	(318 - 593) 195	152	(183 - 863) 481
ID23*	M	Mature	4.5	No	800	1,597	(1,584 - 2,439) 605	437	(21 - 929) 642	834	(749 - 895) 103	583	(318 - 1,311) 702
ID24*	F	Young	3	No	290	1,214	(1,746 - 2,029) 200	590	(440 - 665) 158	719	(1,098 - 1,364) 189	69	(0 - 208) 147
ID26*	M	Young	5	No	180	1,223	(891 - 2,393) 1,062	94	(0 - 263) 186	662	(558 - 1200) 454	546	(174 - 1429) 888

* captured with Tomahawk traps
